# Breast cancer mammospheres secrete Adrenomedullin to induce lipolysis and browning of adjacent adipocytes

**DOI:** 10.1186/s12885-020-07273-7

**Published:** 2020-08-20

**Authors:** Martin Paré, Cédric Y. Darini, Xi Yao, Bérengère Chignon-Sicard, Samah Rekima, Simon Lachambre, Virginie Virolle, Adriana Aguilar-Mahecha, Mark Basik, Christian Dani, Annie Ladoux

**Affiliations:** 1 Université Côte d’Azur, CNRS, INSERM, iBV, Nice, France; 2 Segal Cancer Center, Lady Davis Institute for Medical Research, Sir Mortimer B. Davis Jewish General Hospital, McGill University, Montréal, Québec Canada; 3 Université Côte d’Azur, Pasteur 2 Hospital, Department of Plastic and Reconstructive Surgery, Nice, France; 4 Division of Experimental Medicine, McGill University, Montréal, Québec Canada

**Keywords:** Adipocytes, Breast cancer, Adrenomedullin, Lipolysis, Browning, UCP1

## Abstract

**Background:**

Cancer cells cooperate with cells that compose their environment to promote tumor growth and invasion. Among them, adipocytes provide lipids used as a source of energy by cancer cells and adipokines that contribute to tumor expansion. Mechanisms supporting the dynamic interactions between cancer cells and stromal adipocytes, however, remain unclear.

**Methods:**

We set-up a co-culture model with breast cancer cells grown in 3D as mammospheres and human adipocytes to accurately recapitulate intrinsic features of tumors, such as hypoxia and cancer cell–adipocytes interactions.

**Results:**

Herein, we observed that the lipid droplets’ size was reduced in adipocytes adjacent to the mammospheres, mimicking adipocyte morphology on histological sections. We showed that the uncoupling protein UCP1 was expressed in adipocytes close to tumor cells on breast cancer histological sections as well as in adipocytes in contact with the mammospheres.

Mammospheres produced adrenomedullin (ADM), a multifactorial hypoxia-inducible peptide while ADM receptors were detected in adipocytes. Stimulation of adipocytes with ADM promoted UCP1 expression and increased HSL phosphorylation, which activated lipolysis. Invalidation of *ADM* in breast cancer cells dramatically reduced UCP1 expression in adipocytes.

**Conclusions:**

Breast tumor cells secreted ADM that modified cancer–associated adipocytes through paracrine signaling, leading to metabolic changes and delipidation. Hence, ADM appears to be crucial in controlling the interactions between cancer cells and adipocytes and represents an excellent target to hinder them.

## Background

The tumor microenvironment is crucial for cancer progression and dissemination. It provides nutrients, energy and growth factors to comply with the metabolic requirements of cancer cells. It includes fibroblasts, immune cells, blood vessels and adipocytes. While the importance of cancer associated fibroblasts (CAF) or macrophages for tumor progression was described [1, 2], adipocytes represent a major cell population of the tumor microenvironment that has been neglected for long. In contrast to the healthy tissue context, adipocytes come in direct contact with tumor cells [3, 4] and these interactions were shown to be essential to support malignant progression of invasive carcinomas such as breast, prostate or ovarian cancers [5, 6]. Recent data identified essential drivers for tumor progression expressed when cancer cells were grown in 3D as compared to 2D [7]. However, models mimicking the direct and complex interactions between tumor cells grown in 3D and the surrounding adipose tissue (AT) have not been described so far.

Adipose tissue is widely distributed in the body and enwraps most organs. Two types of AT coexist in adults including white adipose tissue (WAT) which is specialized in energy storage and body weight control through endocrine secretions [8], and brown adipose tissue (BAT) which is able to dissipate energy to properly maintain body temperature. Recently, a new class of adipocytes, called brite (or beige) adipocytes was observed in WAT. These adipocytes derived from white adipocytes, while displaying properties of brown adipocytes, including the expression of the uncoupling protein 1 (UCP1) which uses the mitochondrial proton gradient to produce heat instead of ATP [9–11]. Adipocytes sustain proliferation and dissemination of cancer cells as they provide energy from lipids, as well as growth factors and cytokines [4, 12]. In addition, obesity increases the incidence and worsens the outcomes of several types of cancer such as colorectal, endometrial, prostate, breast and others [13, 14]. However, less information is available on how cancer cells modify adipocytes. Hence, a better knowledge of the interactions between cancer cells and adipocytes is of interest to identify the cross-talk mechanisms supporting tumor expansion.

Breast cancer constitutes a suitable model to study these interactions. The human mammary gland is composed of an epithelial compartment containing ducts and lobules which produce milk during lactation, that is surrounded by connective tissue containing fat lobules [15]. In healthy adults, adipocytes are separated from the epithelial cell compartment by a basement membrane which prevents their interaction. However during post-weaning mammary gland involution, adipocytes and epithelial cells are mixed together [16]. Such a situation is also observed when breast tumors develop. Of note, while adipocytes are generally absent in the core of breast tumors, those located at the invasive front exhibit a lower size as compared to those that are more distal. Similarly, these modifications were described in vitro using co-culture of murine adipocytes and human cancer cells [17]. In addition, a decreased expression of adipocyte markers such as fatty acid binging protein 4 (FABP4), adiponectin and the hormone-sensitive lipase (HSL) were observed in these cells [17, 18]. These cancer-modified adipocytes were called cancer-associated adipocytes (CAA) and they displayed distinct characteristics compared to adipocytes that are not in direct contact with cancer cells. They were reported to play a role in cancer drug resistance [19, 20].

The mechanisms leading to tumor-induced adipocyte transformation still remain enigmatic: the reactivation of the Wnt/β-catenin pathway by Wnt3a secreted by breast tumors may contribute to the development of CAA [21]. Indeed, breast tumors secrete many compounds able to modify the adipocyte phenotype. One of these factors is adrenomedullin (ADM). Its expression was observed in the serum of high-grade breast cancer patients and was indeed correlated with axillary lymph node metastasis [22]. ADM is a 52-amino acid peptide endowed with an extensive repertoire of biological functions including vasodilation and strong hypotensive effects, cellular proliferation, apoptosis modulation or inflammatory regulation. It was originally purified from human pheochromocytoma [23], tumors which are generally surrounded with fat displaying brown/beige phenotype, while more distal adipose depots are unmodified [24, 25]. Its expression is found as well in several other tumor types [26] and tissues pointing out potential autocrine stimulation of tumor cells and paracrine stimulation of tumor surrounding cells. Depending on the cell type, ADM can promote or decrease cell proliferation of both malignant and normal cells [27, 28]. This differential response may result from the complexity of ADM receptors and their presence in distinct tissues. ADM belongs to the calcitonin gene-related peptide (CGRP) family and binds to a G-protein coupled receptor: the calcitonin receptor-like-receptor (CRLR). Binding specificity of ADM to CRLR is conferred by receptor-activity-modifying proteins (RAMPs). ADM binds to CRLR when co-expressed with RAMP 2/3, while CGRP binds to CRLR associated with RAMP1 [29, 30]. ADM binding to the CRLR/RAMP2 complex stimulates the adenylate cyclase leading to the production of cAMP followed by activation of PKA and p38 phosphorylation. ADM expression is strongly up-regulated upon hypoxic conditions through a HIF1-dependent process [31]. Given that solid tumors usually exhibit low oxygen tension, many express and secrete ADM, as measured in high-grade breast cancer patients. ADM was proposed to promote tumor progression though the control of angiogenesis [32] and to accelerate breast cancer bone metastasis [33]. However, the whole impact of ADM on adipocytes has been unheeded so far.

Therefore, we designed a model to assess the interactions between adipocytes and breast cancer cells grown as mammospheres. Here we show that breast cancer mammospheres expressed ADM, while ADM receptors were highly expressed in lipid-laden cells obtained after differentiation of adipose progenitors (AP), including breast APs. Treatment of differentiated APs in vitro, with ADM increased UCP1 expression, resulting in a beige phenotype. Immuno-labeling of breast cancer sections and co-culture experiments of breast cancer mammospheres with lipid-laden cells showed that UCP1 was detected in adipocytes close to the tumor cells. In addition, ADM promoted lipolysis through HSL phosphorylation. Invalidation of *ADM* in MCF7 cells by CRISPR-Cas9 technology, dramatically reduced UCP1 expression in adipocytes, but did not hinder HSL phosphorylation.

Overall, our results show that breast cancer cells grown in 3D directly modify adipocytes in an ADM-dependent manner, promoting then a CAA phenotype.

## Methods

### Reagents

Unless specified otherwise, all reagents were obtained from Sigma (Saint-Quentin Fallavier, France).

Tissue culture media were obtained from LONZA (Levallois-Perret, France) and foetal calf serum (FCS) from Dutscher S.A. (Brumath, France).

### Cell culture

hMADS cells were maintained and differentiated as previously described [34]. They were shown to express a thermogenic signature upon appropriate differentiation conditions [35] and will be further referred to as hMADS-adipocytes.

Breast adipose progenitors were prepared and differentiated as previously described [36].

Adipocytic differentiation was assessed by Oil red O staining [37]. Counter-staining with Crystal violet (0.1% w/v) for 10 min was performed.

Breast cancer cell lines (MCF7 and MDA-MB-231) were grown in DMEM (Phenol red free) supplemented with antibiotics, glutamine and FCS (10%). Sphere formation was carried out on plates coated with agarose in PBS (1% w/v). Cell were plated at a density of 10,000 cells/ml in DMEM medium supplemented with B27 nutrient (Invitrogen), 20 ng/mL EGF and 20 ng/mL FGF2 [38]. Mammospheres were collected by sedimentation and were further used in the experiments.

All cell lines were routinely tested for the absence of mycoplasma.

### Gene expression analysis

Total RNA was extracted using the TRI-Reagent kit (Euromedex, Soufflweyersheim, France) and reverse transcription (RT) was performed using M-MLV reverse transcriptase (Promega, Charbonnieres, France), as recommended by the manufacturers.

All primer sequences are described in the supplementary section. Real-time PCR assays were run on an ABI Prism One step real-time PCR machine (Applied Biosystems, Courtaboeuf, France). Normalization was performed using *36B4* as a reference gene. Quantification was performed using the comparative Ct method.

### Protein expression

Cells were rinsed in ice-cold PBS and whole cell extracts were prepared as described [39]. Briefly, cells were lysed in the cell lysis buffer, sonicated for 10 s and centrifuged at 12000 g for 10 min.

Thirty micrograms of proteins were resolved by SDS–PAGE under reducing conditions and transferred to Immobilon–P membranes (Millipore, Molshiem, France). The detection antibodies listed in supplementary section were used according to the manufacturer’s instructions. The bound primary antibody was detected by horseradish peroxidase-conjugated secondary antibody and visualized using an ECL detection kit (Millipore, Molsheim, France). Chemiluminescence was observed using a molecular imager ChemiDoc XRS system (Bio-Rad, Marne la Coquette, France). Band intensity was quantified using Bio-Rad Quantity One software.

### *UCP-1* expression analysis using a reporter system

hMADS cells were infected with a lentivirus containing the plasmid, pLV.ExBi.P/Puro-hUCP1promoter-Luc (firefly)-T2A-hrGFP, which expresses luciferase and GFP under the human UCP1 promoter (4148 bp) [40] and the cells were further selected with puromycin.

Upon stimulation of the cells, GFP expression was quantified using ImageJ on images recorded with identical parameters. GFP expression levels were compared to those measured in unstimulated cells.

### Immunocytochemistry

Cells were seeded on glass coverslips and treated as indicated in the legend. Labelling was performed as previously reported [41] and the antibodies used are listed in the supplementary data. They were revealed with the appropriate secondary antibody coupled to Alexa Fluor (1/1000 v/v). Unspecific signal was evaluated for each antibody using a control condition without primary antibody and a non-specific antibody. Six to ten representative fields were examined for each condition. Images were taken on a Zeiss Axio Observer microscope with an EC Plan Neofluar 40X (NA 1.3) oil objective using AxioVision 4.8.2 software. Analysis was performed using Fiji [42].

### Cytochemistry & immunohistochemistry

Formalin-fixed paraffin-embedded (FFPE) tissues were cut at 4-μm and placed on SuperFrost/Plus slides (Fischer) before IHC processing. For histological analyses, sections were subjected to hematoxylin and eosin or Picro-Sirius red staining.

Immunostaining of UCP1 was performed using a rabbit anti-UCP1 (1/200; Abcam) revealed by a goat anti-rabbit-biotynylated (1/500 Vector laboratories). Slides were counterstained with hematoxylin and eosin. Image acquisition was performed using the Vectra Polaris slide scanner (40X).

### ADM gene invalidation

Cells were transfected with plasmid expressing Cas9 nuclease and two guide RNAs targeting the *ADM* gene (supplementary Fig. 7). The CRISPR kit used for constructing multiplex CRISPR/Cas9 vectors was a gift from Takashi Yamamoto (Addgene kit # 1000000055) [43].

The CRISPR/Cas 9 procedure induced a 584 base pairs deletion in the genomic DNA accompanied by a shift in the open reading frame. PCR amplification of the genomic region targeted by the CRISPR/Cas9 procedure produced 535 bp or 1119 bp amplicons for targeted clones and WT genomic DNA respectively. The crude PCR product was purified and sequenced for 10 clones. Deletion was further confirmed at the RNA level, by RT-PCR. In this case, 366 or 569 bp amplicons were observed in modified or WT clones respectively.

### Statistical analysis

The results are shown as mean + standard error of the mean (SEM), with the number of experiments indicated. Statistical significance was determined by *t-*tests using Micrococal Origin 6.0 (Micrococal Software, Northampton MA) or BiostaTGV (INSERM and Sorbonne University, PARIS, France). Probability values < 0.05 were considered statistically significant and are marked with a single asterisk, double asterisks (< 0.01) and triple asterisks (< 0.001).

## Results

### Setting-up a model to mimic the interactions between mammospheres and peritumoral adipocytes

**Fig. 1:**
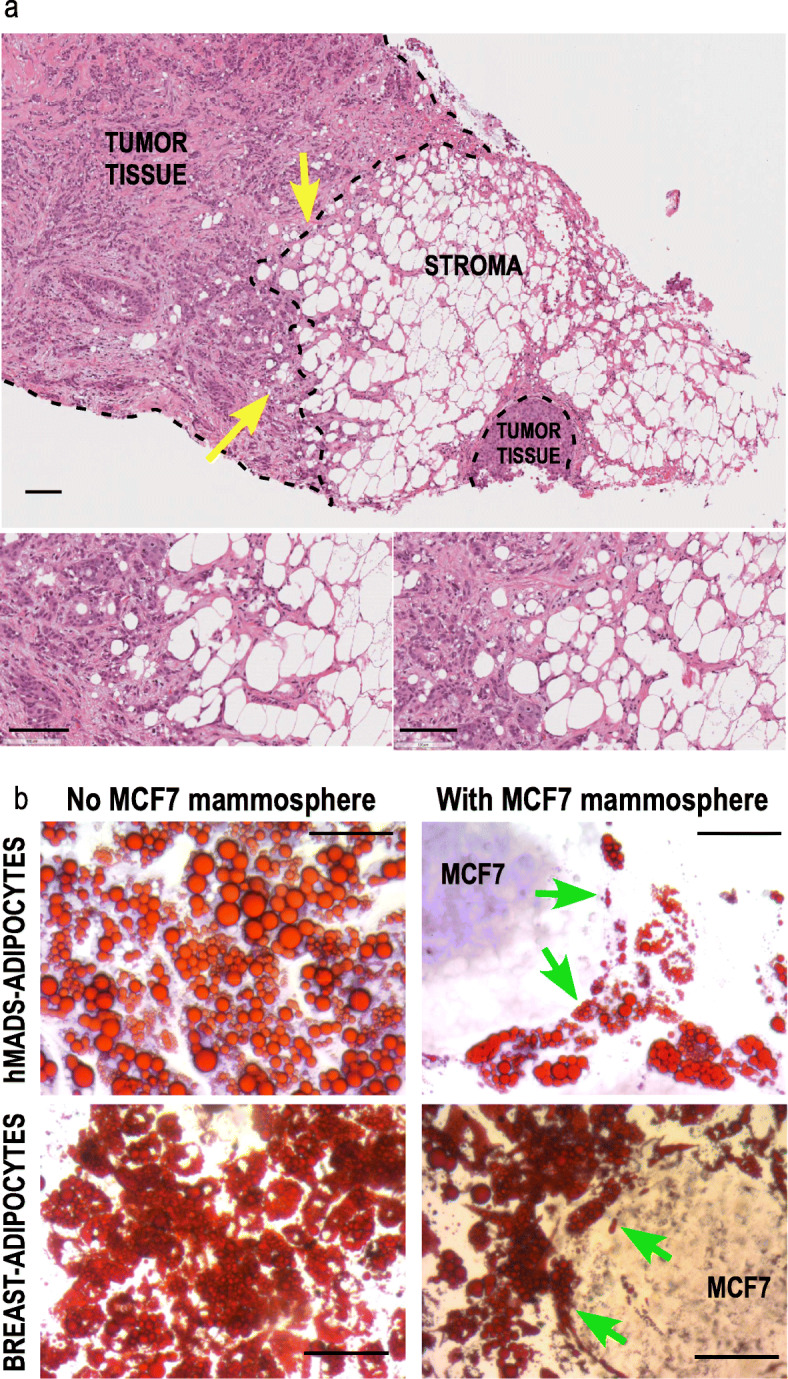
Morphology of adipocytes in contact with tumor cells or close to MCF7 mammospheres. **a** Histological analysis of human breast tumor section containing adipocytes. Histology of breast tumor sections was revealed by hematoxylin and eosin staining. Adipocytes with small lipid droplets (yellow arrows) are preferentially observed close to the tumor cells, while those which are more distal display larger lipid droplets. (scale bar 100 μm). The two insets correspond higher magnification view of the zones indicated by the arrows. **b** hMADS-adipocytes or breast-adipocytes were cultured in differentiation medium for 15 days. MCF7 cells were grown as mammospheres for 7 days. Co-culture of the 2 cell-types was performed for 2 days. Oil-Red O staining reveals that the size of the lipid droplets is heterogeneous, although smaller in adipocytes adjacent to the mammospheres (green arrows). Crystal violet was used to counterstain the MCF7 cells in presence of hMADS-adipocytes. Magnification X20, scale bar 100 μM

Histological sections of breast tumors show that adipocytes in proximity to cancer cells have smaller lipid droplets than those more distal, which is not observed in healthy breast tissue (Fig. 1a & Supp. Figure 1). To better mimic this situation, we grew breast cancer cell lines as mammospheres that were added to hMADS- or breast-adipocytes cultures to visualize cell to cell interactions. We observed that in both cases, lipid droplets were smaller in adipocytes close to the mammospheres compared to more distal adipocytes or to the cultures without mamospheres (Fig. 1b & Supp. Figure 1), mimicking the morphology of adipocytes observed on histological sections.

### Brown adipocytes are observed close to tumor cells

**Fig. 2:**
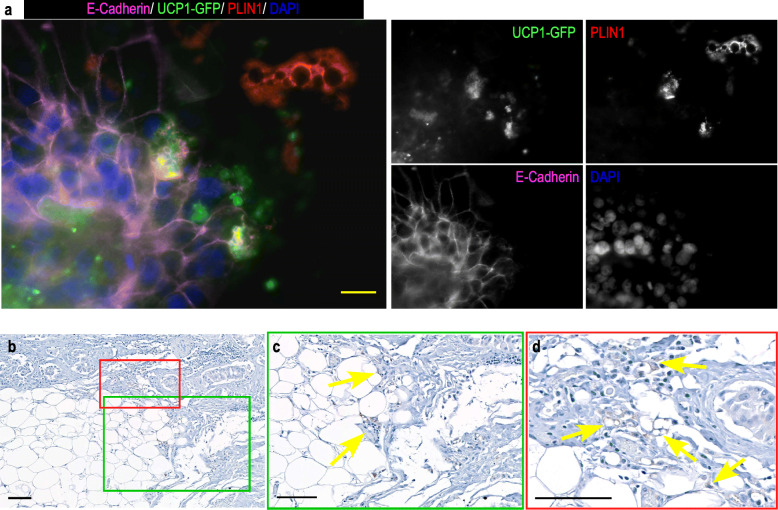
UCP1 expression in adipocytes close to tumor cells. **a** / MCF7 cells were grown as mammospheres for 7 days. They were co-cultured on a monolayer of hMADS cells expressing GFP under the control of UCP1 promoter which had been differentiated for 14 days. Co-culture of the 2 cell types lasted for 4 days. PLIN1 (red, labelling the lipid droplets) and E-Cadherin (pink, labelling the MCF7 mammospheres) expressions were visualized using specific antibodies. GFP expression was visualized in green. DAPI was used to label the nuclei (Blue). The fluorescence recorded for each channel is shown in separated images. The adipocytes adjacent to the mammospheres express both GFP and PLIN1, while those more distant from the mammospheres express PLIN1 only. Magnification 40X, scale bar 20 μM. **b**, and insets **c** & **d** UCP1 expression in patient breast tumor sections was detected using immunohistochemical labeling with anti-UCP1 antibody (arrows). The immunostains are shown in brown. Red and green frames are used to localize UCP1 expression in the section. Magnification 10X (**b**), 20X (**c**) & 40X (**d**), scale bar 100 μM

This result prompted us to analyze the MCF7-induced metabolic changes in hMADS-adipocytes using hMADS-adipocytes expressing GFP under the control of *UCP1* promoter. We noticed that GFP was preferentially expressed in adipocytes close to the mammospheres. Indeed, most, but not all, adipocytes in contact with the mammospheres were expressing both GFP and PLIN1, a protein found in the membrane of adipocyte lipid droplets. hMADS-adipocytes in a more distal position expressed PLIN1 only (Fig. 2a). This indicated that mammospheres induced adipose browning of adipocytes in contact or in close proximity. MDA-MB-231 mammospheres produced a similar result (Supp. Figure 1). Mammospheres induced UCP1 expression in breast-adipocytes (Supp.Figure 2a). Note that MCF7 cells displayed a positive staining for UCP1 (red arrow) that was confirmed by western blot using a different antibody (Supp. Figure 2b).

In parallel, IHC analyses of breast tumor histological sections showed that some adipocytes located close to tumor cells and presenting small lipid droplets, displayed UCP1 labelling (Fig. 2b-c-d & Supp. Figure 2c). We analyzed sections from HER2 positive tumors and three out of nine patients displayed adipose-specific UCP1 labelling. No correlation with the presence of estrogen or progesterone receptors could be established. This result indicated that adipose browning occurred also in some but not all tumors in vivo.

### Expression of ADM and its receptors

#### ADM is produced by mammospheres

**Fig. 3:**
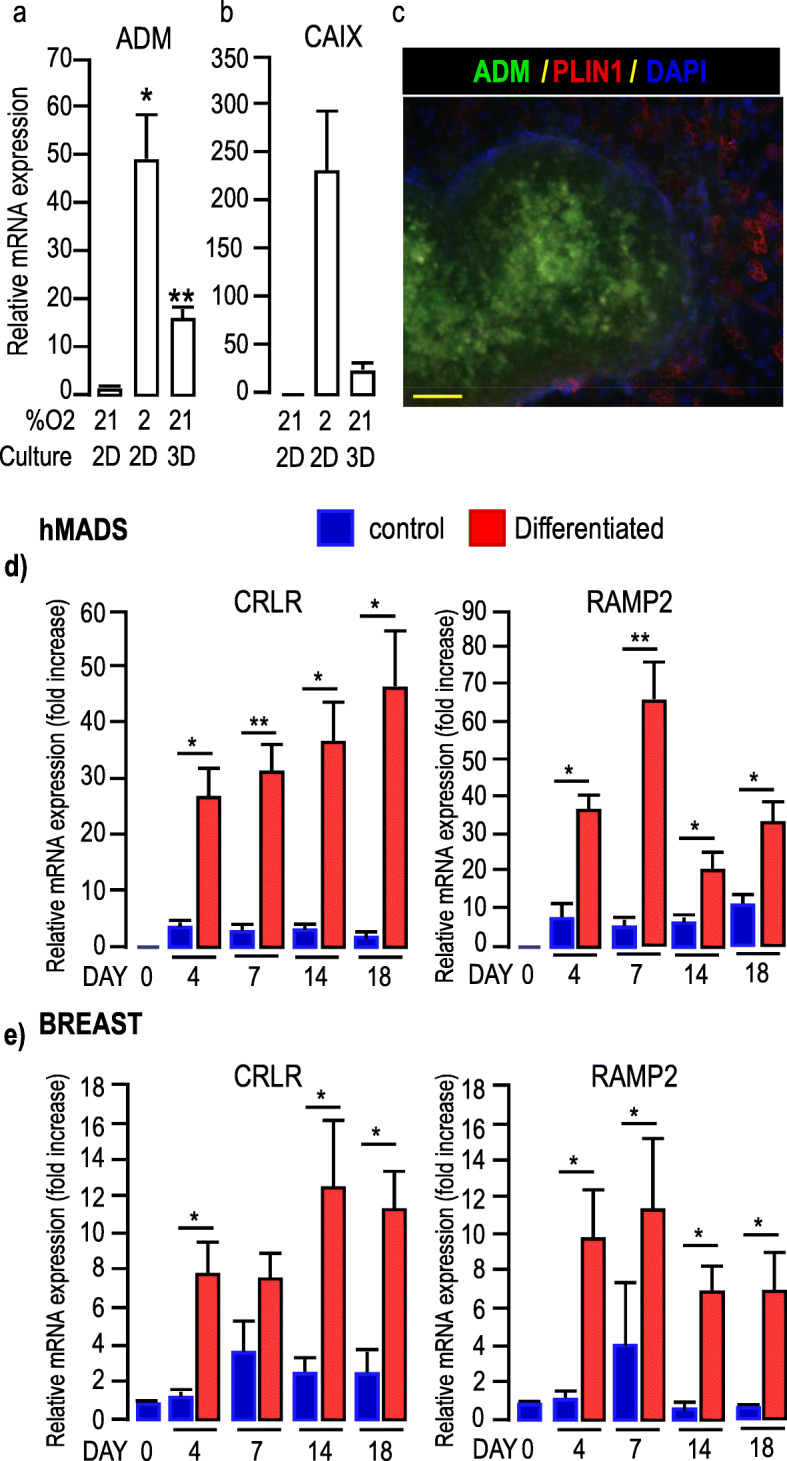
Expression of ADM in MCF7 cells and ADM receptors in adipocytes. **a-b,** Expression of *ADM* and *CA IX* in MCF7 cells cultured in different conditions. Expression of *ADM* and *CA IX* was assessed by real-time RT-PCR and normalized for the expression of *36B4* mRNA. Expression was measured in cells grown as 2D monolayers in the presence of 21 or 2% O2 for 48 h or as mammospheres for 7 days. The means ± SEM were calculated from three independent experiments, with determinations performed in duplicate (*p < 0.05, ** *p* < 0.01). **c,** Expression of ADM in mammospheres. MCF7 cells were grown as mammospheres for 7 days. Expressions of ADM (green) and PLIN1 (red, to label adipocytes) are shown. DAPI was used to label the nuclei (Blue). The fluorescence recorded for each channel is shown in separated images (see Supp. Figure 3). Note that ADM was not expressed in adipocytes, but in mammospheres. Scale bar: 50 μm. **d-e** ADM receptors expression in hMADS (**d**) and breast-adipocytes (**e**). Time course for the expression of *CRLR* and *RAMP2,* was assessed by real-time RT-PCR and normalized to the expression of *36B4* mRNA. Expressions were measured in cells that received (red bars) or did not receive (blue bars) the differentiation cocktail for the indicated number of days. The means ± SEM were calculated from three independent experiments, with determinations performed in duplicate (*p < 0.05, ** p < 0.01)

We hypothesized that the adipose metabolic conversion may result from a hypoxia-induced molecule produced by the mammospheres and therefore, we focused on ADM. Upon binding to its receptor, it elevates cAMP levels [31] which activate a crucial pathway inducing adipose browning. Growing MCF7 cells under hypoxia (i.e. 2% O2) or as 3D mammospheres significantly increased *ADM* mRNA levels compared to 2D cells maintained under 21% of oxygen (Fig. 3a). In parallel, expression of the hypoxic marker carbonic anhydrase IX (*CAIX*), was increased (Fig. 3b). Intermedin (i.e. adrenomedullin2) was mainly expressed in MCF7 cells grown in 2D, but not in mammospheres or in cells maintained under hypoxia (Supp. Figure 3a). ADM expression was further confirmed in mammospheres by immunocytochemistry using a specific anti-ADM antibody (Fig. 3c & Supp. Figure 3b). It was more elevated in the center of the spheres which corresponded to the most hypoxic zones.

Thus, when grown as mammospheres, breast cancer cells expressed ADM, a hypoxia-inducible gene that was not detected in 2D cultures.

#### ADM receptors are expressed in adipocytes

Analysis of ADM receptors expression in hMADS cells revealed that both *CRLR* and *RAMP2* mRNA were hardly detectable in undifferentiated cells, while their expressions increased significantly during the time course of differentiation. After 18 days of differentiation the expression levels for both *CRLR* and *RAMP2* were raised 40 times as compared to undifferentiated cells (Fig. 3d). Note that *RAMP1*, which is also able to associate with *CRLR* to form a CGRP, but not ADM, receptor, was preferentially expressed in undifferentiated cells (Supp. Figure 4a). In addition, the expression of Receptor Component Protein (RCP) which associates with the CRLR-RAMP2 complex was detected similarly in both undifferentiated and differentiated cells (Supp. Figure 4a), indicating that this complex can couple to Gs and activate the cAMP-dependent intracellular signaling pathway. Similar results were observed for breast- adipocytes (Fig. 3e & Supp. Figure 4b). Note that neither *CRLR* nor *RAMP2* mRNA were present in MCF7 mammospheres, therefore preventing any autocrine effect of ADM (Supp. Figure 4c).

### ADM induced UCP1 expression

**Fig. 4:**
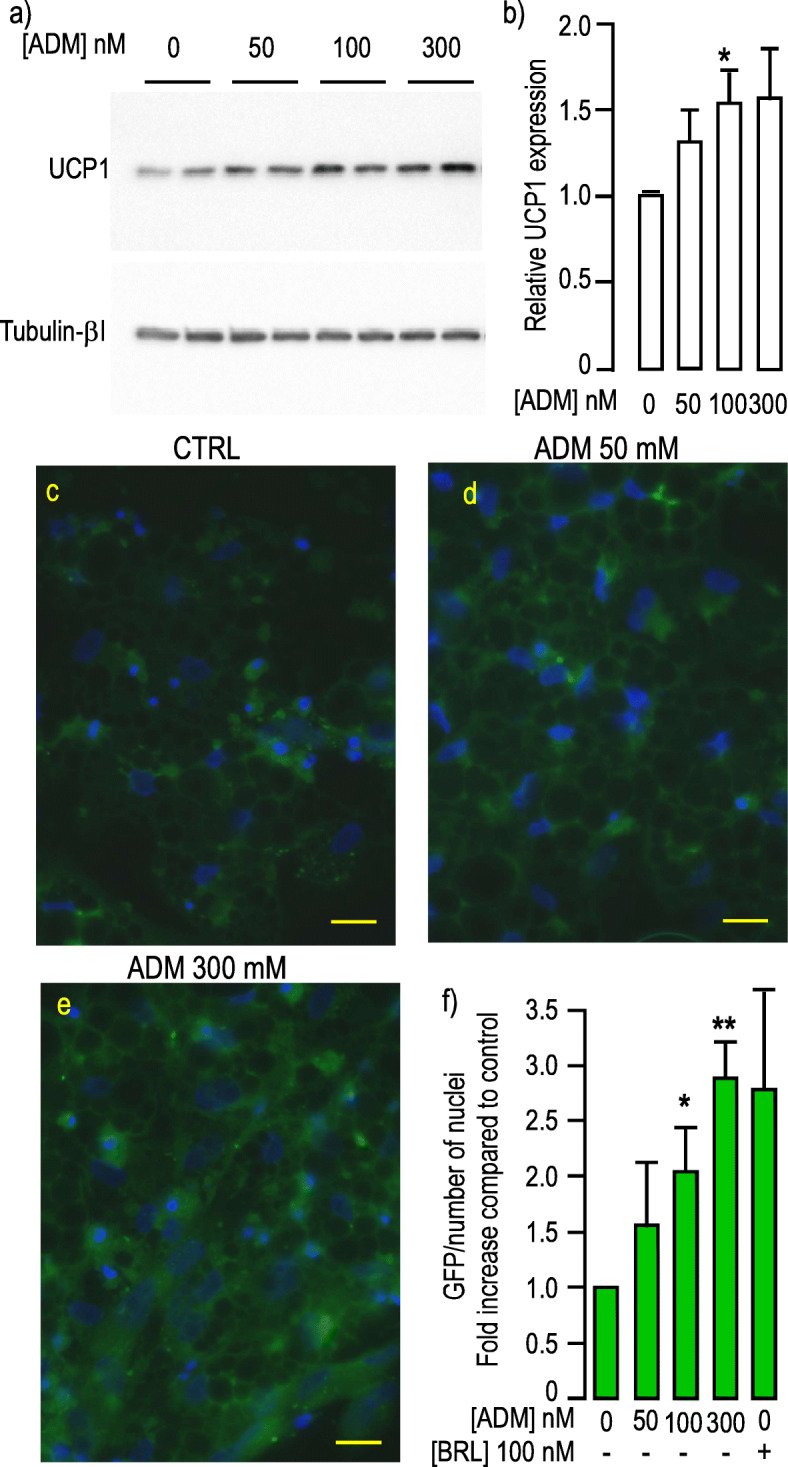
ADM induced UCP1 expression. **a**, ADM induced UCP1 in a dose dependent manner. Protein expression was measured in hMADS-adipocytes grown in differentiation medium for 17 days. They were stimulated by increasing doses of ADM for 24 h. Expressions of UCP1 (upper panel) and Tubulin-βI (lower panel) used as a loading control were analyzed by Western blot using specific antibodies. Representative Western blots are shown. Full-length blots are presented in Supplementary Figure 9. **b**, Quantification of the signals. Protein expression was quantified using Quantity One Program and compared to the expression of Tubulin-βI. The means ± SEM were calculated from four independent experiments (*p < 0.05). **c-e,** Transcriptional activation of *UCP1* promoter in response ADM. GFP expression was driven by the human UCP1 promoter in hMADS cells differentiated for 14 days. GFP fluorescence was determined after 4 more days of incubation with the indicated concentration of ADM. Control condition corresponds to cells that did not receive ADM. All images were recorded with the very same parameters. (Scale bar: 20 μM). **f,** Quantification of the signals. Means were calculated from 3 independent experiments performed in duplicate on at least 6 distinct recordings for each coverslip. The fluorescence signal measured as “raw integrated density” was divided by the number of nuclei present in the microscopic field. Rosiglitazone (BRL) was used as a positive inducer of browning. (*p < 0.05, **p < 0.01)

We next challenged hMADS-adipocytes with increasing concentrations of ADM and measured UCP1 expression. UCP1 was significantly increased when cells were treated with ADM concentrations between 100 and 300 nM (Fig. 4a-b). This result was confirmed using cells expressing the UCP1-GFP reporter system. After 14 days of differentiation, cells were further treated for 4 days with increasing doses of ADM. While untreated cells expressed a weak GFP signal, GFP fluorescence raised in a dose-dependent manner (Fig. 4c-f). Quantification of the signals revealed that ADM (300 nM) induced a 2.6-fold increase in fluorescence intensity compared to cells that did not receive any treatment. Altogether our results indicate that ADM induced UCP1 expression in hMADS-adipocytes.

### ADM induced lipolysis in hMADS and breast-adipocytes

**Fig. 5:**
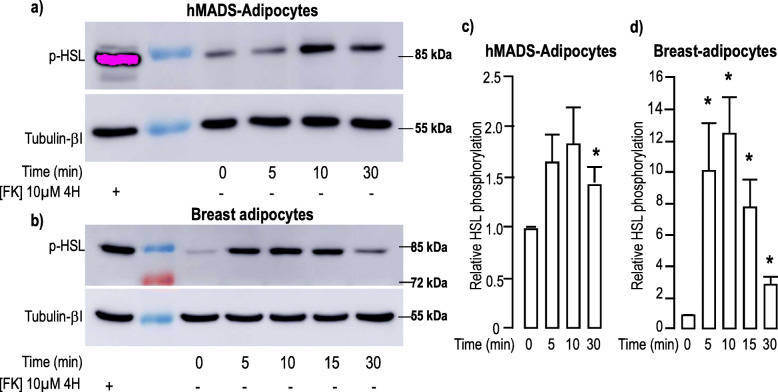
ADM induced phosphorylation of HSL. **a-b,** ADM increased phosphorylation of HSL. Protein expression and phosphorylation were measured in hMADS-adipocytes (panel a) or breast adipocytes (panel b) grown in the differentiation medium. They were stimulated with 100 nM ADM for the indicated time. Expressions of pHSL (upper panel) and Tubulin-βI (lower panel) used as a loading control were analyzed by Western blot using specific antibodies. A positive control condition with cells stimulated for 4 h with forskolin is shown. Representative Western blots are shown. Full-length blots are presented in Supplementary Figure 9. **c-d,** Quantification of the signals. Expression and phosphorylation of the proteins were quantified using the Quantity One Program and compared to the expression of Tubulin-βI. The means ± SEM were calculated from four independent experiments. (*p < 0.05)

Next, we looked for additional ADM-induced effects on adipocytes and measured the time course for ADM-induced HSL phosphorylation (pHSL) which occurs upon PKA stimulation. Indeed, a 10 min-treatment of hMADS-adipocytes with 100 nM ADM induced a 2-fold increase in pHSL (Fig. 5a&c). Similar treatment applied to breast-adipocytes induced a higher level of pHSL (up to 10 times) (Fig. 5b&d). In co-culture experiments, phosphorylation of HSL was observed in hMADS- or breast-adipocytes located close to MCF7- (Supp. Figure 5a-b) or MDA-MB-231- (Supp. Figure 5c) mammospheres. Phosphorylated HSL co-localized with PLIN1. This observation was consistent with the translocation of the enzyme from the cytosolic compartment to the lipid droplet. Note an intense labeling within the mammospheres which likely corresponds to the MCF7-induced modifications of the adipocytes below them. No pHSL labeling was detected in breast cancer mammospheres (Supp. Figure 5d).

HSL phosphorylation occurred concomitantly with a slight, but significant, increase in p38-MAPK phosphorylation that was detected after 5–10 min. and remained observable after 30 min of incubation with ADM (Supp. Figure 6).

Thus, ADM induced phosphorylation of HSL and p38-MAPK both in hMADS- and breast-adipocytes that may favor free fatty acid release to provide energy to cancer cells.

### ADM invalidation in MCF7 cells and its consequences in adipocytes

#### Invalidation of ADM

**Fig. 6:**
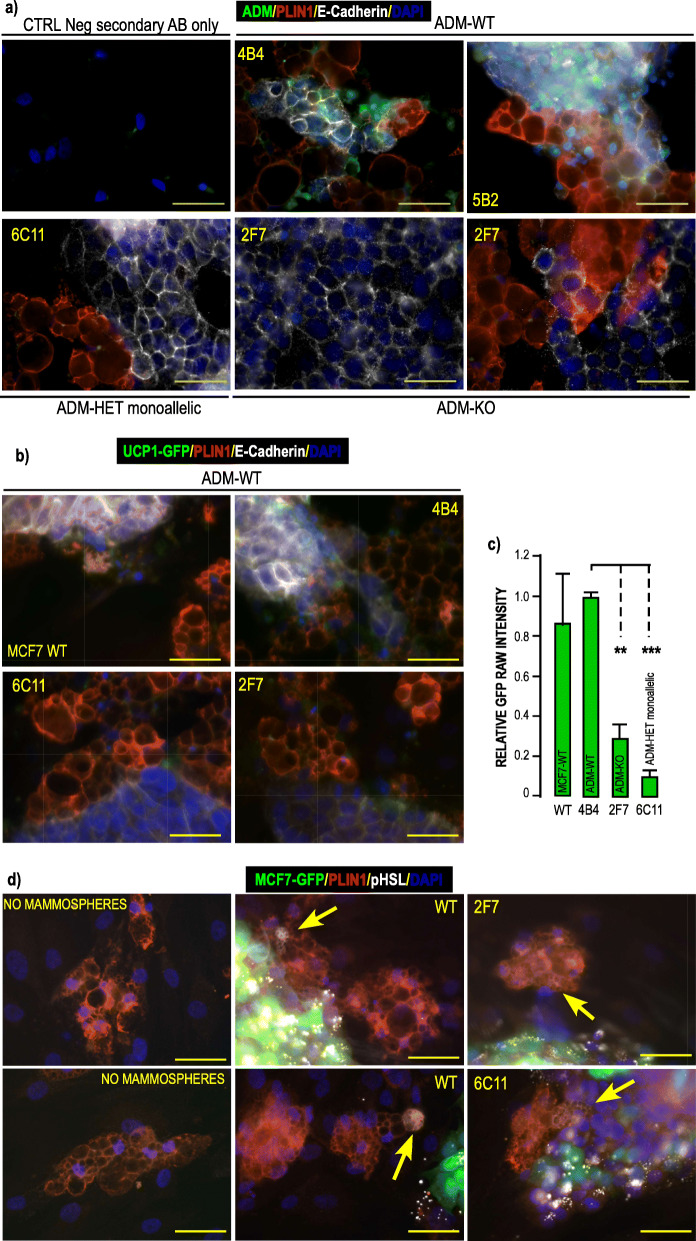
Analysis of ADM mutated clones obtained by CRISPR Cas9 technology. **a** *ADM expression in mutated clones obtained by CRISPR Cas9 technology****.*** WT MCF7, ADM-Het and ADM-KO MCF7 cells were grown as mammospheres for 7 days. They were co-cultured on a monolayer of hMADS cells that had been differentiated for 14 days. Co culture of the 2 cell types lasted for 4 days. ADM (green) PLIN1 (red) and E-Cadherin (white) expressions were visualized using specific antibodies. DAPI was used to label the nuclei (Blue). Images were recorded using the very same settings for ADM signal (i.e. 300 m seconds.). Magnification 40X, scale bar 50 μM. **b** *ADM mutated clones are less efficient at inducing UCP1 expression in hMADS- adipocytes.* WT-MCF7, ADM-Het and ADM-KO MCF7 cells were grown as mammospheres for 7 days. They were co-cultured for 4 days on a monolayer of hMADS cells expressing GFP under control of the UCP1 promoter that had been differentiated for 14 days. PLIN1 (red) and E-Cadherin (white) expressions were visualized using specific antibodies. GFP expression was visualized in green. DAPI was used to label the nuclei (Blue). Images were recorded using the very same settings. Magnification 40X, scale bar 50 μM. GFP signals were quantified using ImageJ software and compared to signals obtained in the presence of the WT MCF7 cells. **c** *Quantification of the signals.* Means were calculated from 3 independent experiments performed in duplicate on at least 3 distinct recordings for each coverslip. The fluorescence signal measured as “raw integrated density” and compared to the signal obtained with WT-MCF7 cells. (**p < 0.01, ***p < 0.001). *d) ADM mutated clones induced HSL phosphorylation in hMADS- adipocytes.* WT MCF7, ADM-Het and ADM-KO MCF7 expressing GFP cells were grown as mammospheres for 7 days. They were co-cultured for 3 days on a monolayer of breast adipocytes that had been differentiated for 21 days. PLIN1 (red) and pHSL (white) expressions were visualized using specific antibodies. GFP expression was visualized in green. DAPI was used to label the nuclei (Blue). Images were recorded using the very same settings. Arrows indicate pHSL labelling in cells expressing PLIN1. Magnification 40X, scale bar 50 μM. Single channels pictures are presented in supplementary figure 8

To invalidate *ADM* in MCF7 cells, we set out to knock out the two alleles of the gene using the CRISPR/Cas9 technology. After transfection of the RNA guide/Cas9 expressing construct that contained two guide RNAs after the U6 promoter (Supp. Figure 7a) we analyzed 128 clones. We obtained 14.8% of heterozygous clones (referred as ADM-HET) and only 1 clone displaying deletion on both alleles (referred as ADM-KO) (Supp. Fig. 7b.). Comparison of the ADM-Het and ADM-KO sequences to the wild type (WT) sequence (Supp. Figure 7c) revealed that the deletion of the expected zone was associated with a single nucleotide insertion after the cutting site of the first guide RNA, leading then to a shift in the open reading frame. The genomic sequences of the mutated alleles in ADM-Het and ADM-KO clones were identical, except for one ADM-Het clone (clone 6-C11) that had an additional 20 bp deletion in the 5′-region targeted by the first guide RNA. For all selected clones, the shift in the open reading frame prevented any functional ADM protein translation (Supp. Fig. 7d). In addition, all the cleavage sites of pre-pro-ADM were lost, thus precluding the formation of a mature peptide. Surprisingly, analysis of the *ADM* mRNA indicated that most of the ADM-HET clones displayed monoallelic expression of the mutated allele that lasted with time (Supp. Figure 7b), indicating that they may behave as ADM-KO clones. In line with this observation, monoallelic expression of the estrogen receptor was also described in breast cancer [44]. ADM peptide could not be detected by ICC in mammospheres from ADM-KO clones (Fig. 6a & Supp. Figure 8).

#### Absence of ADM in MCF7 cells impaired UCP1 expression

We compared UCP1 expression in UCP1-GFP hMADS-adipocytes maintained with either WT MCF7 cells or clones grown as mammospheres: ADM-WT (4-B4, 5-B2), ADM-HET (6-C11), ADM-KO (2-F7).

hMADS-adipocytes adjacent to WT-MCF7 or ADM-WT mammospheres expressed GFP, as expected. Interactions of ADM-KO or monoallelic ADM-Het clones revealed that GFP expression was impaired in hMADS adipocytes (Fig. 6b). Quantification indicated that 2F7-mammospheres reduced the GFP signals by 70%, while the decrease reached 90% with the 6C11 clone (Fig. 6c).

Thus *ADM* deletion strongly reduced the browning of lipid-laden cells adjacent to mammospheres.

#### Absence of ADM in MCF7 cells did not abolish HSL phosphorylation

We evaluated HSL phosphorylation in breast adipocytes incubated with or without ADM-invalidated clones. No HSL phosphorylation was detected in the absence of mammospheres while it was observed in adipocytes adjacent to WT as well as ADM-invalidated mammospheres (Fig. 6d).

This indicated that in addition to ADM, other molecules secreted by the mammospheres, were involved in this process.

## Discussion

In this study, we set up a model to better analyze the cross-talk between breast cancer cells and adipocytes. We showed that breast cancer mammospheres impacted preferentially adipocytes located close to them, while distal adipocytes were less affected. They increased lipolysis and induced a shift towards a beige phenotype. ADM was secreted by mammospheres and it promoted both modifications in adipocytes, which are consistent with the occurrence of a CAA phenotype.

Breast cancer cells grown as mammospheres display a hypoxic environment crucial for tumor progression and our in vitro model is appropriate to study how adipocytes are impacted. Indeed, mammospheres expressed CAIX, a hallmark of hypoxic regions [45]. Thus, they produced hypoxia-inducible peptides which are secreted in the tumor core but are absent from classical monolayer cultures. We observed that tumor mammospheres modified adipocytes, as reported with 2D cultures. However, a preferential effect on the lipid-laden cells adjacent to the tumor was seen, while distant adipocytes were less affected. Such a situation was observed on histological sections: morphological differences exist between the adipocytes present at the peritumoral border compared to those which are distal to the tumor cells [5]. This may result from a direct contact between tumor cells and the adipocytes, but also from a concentration gradient of molecules secreted by the tumor. Cancer cells promote lipolysis in surrounding adipocytes. We detected phosphorylated-HSL mainly in the lipid droplets membranes of adipocytes close to tumor cells. Then the released lipids, including free fatty acids (FFAs), help to promote tumor expansion [17, 46] upon mitochondrial β-oxidation [4], by producing signaling lipids or lipids incorporated into the membranes of rapid-growing malignant cells. In addition, a beige phenotype, through UCP1 expression, was observed. It mainly occurred in CAA adjacent or close to tumor cells that displayed small lipid droplets and this was also observed in histological sections from patients. Thus, our model better recapitulated the adipose modifications observed in breast cancer tissue sections and pointed out that mammospheres alone (i.e. in the absence of other cell types, such as immune cells, present in the tumor environment) were able to remodel adipocytes.

The murine adult breast AT is composed of white adipocytes that retain high plasticity potential. During pregnancy and lactation they transdifferentiate into adipocytes suited for milk production [47]. Note that the presence of brown/beige adipocytes within breast fat pads was described during the early stages of murine mammary gland development [48, 49]. Multilocular adipose regions displaying a positive staining for UCP1, were observed in Brca1 mutant mice [50] and the presence of brown/beige adipocytes surrounding breast cancer cell lines and breast PDX xenografts was detected indicating that the tumor may favor/induce metabolic changes in the surrounding adipocytes. Although brown fat is not a normal constituent of human adult breast, human mammary hibernomas were described [51, 52]. The presence of brown/beige adipocytes was proposed to be beneficial for tumor expansion as depletion of the UCP1 positive cells significantly reduced tumor growth [53], hence tumor-induced browning was recognized as an important target to propose novel therapeutic strategies to control breast tumor progression. We observed that estrogen positive MCF7 cells, triple negative MDA-MB-231 cells and HER2+ tumors induced browning, indicating that it could not be related to a precise breast tumor type. However, the mechanisms underlying these modifications are not yet well understood. It was proposed that within xenografts, beige progenitors, recruited from an unidentified source, differentiated into beige adipocytes [53]. Another possibility is the trans-differentiation of white adipocytes into UCP1-expressing cells. Although a better knowledge of the mammary adipose progenitors deserves attention, both hypotheses include a specialization of cells to acquire beige characteristics. Few biological inducers of this process produced by cancer cells have been reported so far, one of such factors being Wnt3a [21]. Inflammatory cytokines produced by immune cells, such as IL6 or TNFα, may also induce adipocyte browning/beiging. However, their contribution is unlikely in our simplistic model as only the tumor and lipid laden cells differentiated from APs interact.

Breast tumor mammospheres secreted ADM, which is known to be highly produced in hypoxic environments of several tissues, including adipose tissue [31, 54, 55]. Hypoxia is a prevailing feature of many tumors with hypoxic niches located preferentially in the center of the tumor. In good agreement, we observed that ADM immuno-labeling was higher in the center of MCF7 mammospheres. We showed that ADM induced UCP1 expression and beige adipocyte formation. ADM was also able to activate HSL and promote lipolysis which has been described as a paramount process to fuel tumors and promote adipocyte browning/beiging. ADM directly increased HSL phosphorylation. Previous report in line with our observations, described that inflammation of the human AT stimulated the ADM-induced lipolysis [56]. But HSL phosphorylation occurred in adipocytes close to ADM-KO-MCF7 mammospheres indicating that ADM was not the only inducer of lipolysis. Then, multiple molecules were involved in tumor-induced lipolysis [57]. In contrast, thanks to our co-culture model, we observed that ADM was the major inducer of adipose browning. Indeed, adipocytes expressed less UCP1 in presence of ADM-KO mammospheres.

So far, intermedin which belongs to the same peptide family as ADM was described to induce beiging of white adipose tissue [58, 59]. However, MCF7 mammospheres expressed less intermedin than ADM, thus restraining its ability to activate ADM receptors. A similar expression profile was found for PTHrP which is reported to induce browning and cancer cachexia in mouse xenografts models [60].

Besides the modification of adipocyte metabolism, the role of ADM in cancer progression may be multifactorial. An autocrine role of ADM to promote tumor growth through control of proliferation, or inhibition of apoptosis has been described in breast cancer cell lines [61]. In MCF7 cells, we did not observe ADM receptor expression, ruling out such regulation in our model. The ability of ADM to change the tumor microenvironment via paracrine secretion was mainly mentioned in an angiogenic context. ADM promotes proliferation and migration of endothelial cells, so its role in stimulating tumor angiogenesis has been studied using several in vitro or xenograft and knockout mouse models [28, 62, 63]. ADM was also shown to reduce the activity of the immune system by decreasing cytokine secretion [64] which leads to a defective anti-tumor immuno-monitoring. Thus, ADM is endowed with properties that can efficiently contribute to activation of distinct pathways promoting tumor growth: lipolysis and browning. Lipolysis certainly provides FFAs as an energy source for tumor cells as already reported [17, 46]. In this regard, the lipolysis-induced FFAs release may not just serve as an “energy substrate”. FFAs may participate in remodeling the microenvironment. They were shown to facilitate breast cancer invasion through induction of plasminogen activator inhibitor-1 (PAI-1) and inhibition of fibrin degradation, facilitating then cancer invasion and metastasis [65]. The exact role of adipose browning for tumor progression remains to be established. One may hypothesize that it promotes carcinoma progression as brown adipocytes depletion reduces tumor growth [53]. Paracrine interactions between cancer cells and their environment including adipocytes mainly occur at the invasive front of tumors. This can explain why metabolic reprogramming of adipocytes occurs nearby cancer cells and why it has not been detected in two dimensional co-cultures of cancer cells and adipocytes.

## Conclusion

Altogether, our results bring new information regarding the complex interactions between adipocytes and breast tumor cells. We identified that cancer cells in mammospheres, through ADM secretion, were able to modify the phenotype of adipocytes. However, further investigations are required to understand the precise role of the UCP1-expressing cells and the importance of the adipose metabolic conversion induced by cancer cells during malignancy.

## Supplementary information

**Additional file 1.**

## Data Availability

All data generated and/or analysed during this study are included in this published article (and its supplementary information files).

## Abbreviations

ADM: Adrenomedullin
AP: adipose progenitors
AT: adipose tissue
BAT: brown adipose tissue
CAA: cancer-associated adipocytes
CAIX: carbonic anhydrase IX
cAMP: cyclic AMP
Cas9: CRISPR associated protein 9
CRISPR: Clustered Regularly Interspaced Short Palindromic Repeats
CRLR: calcitonin receptor-like-receptor
EGF: Epidermal growth factor
FABP4: fatty acid binding protein 4
FFAs: free fatty acids
FFPE: Formalin-fixed paraffin-embedded
FGF2: fibroblast growth factor 2
GFP: green fluorescent protein
hMADS: human Mesenchymal Adipose-Derived Stem cells
HIF1: hypoxia-inducible factor 1
HSL: hormone-sensitive lipase
IHC: immune histo chemistry
M-MLV: Moloney Murine leukemia virus
PAI-1: plasminogen activator inhibitor-1
PKA: protein kinase A
PBS: Phosphate buffered saline
PCR: Polymerase chain reaction
PLIN1: Perilipin 1
RAMPs: receptor-activity-modifying proteins
RT: reverse transcription
UCP1: uncoupling protein 1
WAT: white adipose tissue
