# Supplementary material for: Breast cancer mammospheres secrete Adrenomedullin to induce lipolysis and browning of adjacent adipocytes
Source: BMC Cancer. 2020 Aug 20;20:784. doi: 10.1186/s12885-020-07273-7 (PMC7441622; doi:10.1186/s12885-020-07273-7)

SUPPLEMENTARY INFORMATION

Breast cancer mammospheres secrete Adrenomedullin to induce lipolysis and browning of adjacent adipocytes.

Martin PARE[1], Cédric DARINI[2], Xi YAO[1], Bérengère CHIGNON-SICARD[3], Samah REKIMA[1], Simon LACHAMBRE[1], Virginie VIROLLE[1], Adriana AGUILAR-MAHECHA[2], Mark BASIK[2,4], Christian DANI[1] and Annie LADOUX[1].

Supplementary Information

Sequences for the primers used in this study

**Gene**	**Forward primer**	**Reverse primer**
ADM	5'- CCAGAGCATGAACAACTTCC -3’,	5’- GTCCTTGTCCTTATCTGTGAAC -3’,
CRLR	5’- CACCCACGAGAAAGTGAAG -3’	5’- TGCCAAGCGAGATAAGCAG -3’
RAMP1	5’- CTGGCACATGGCGGAGAAG-3’	5’- CTGAAGTAGCGGCCATGCAC -3’
RAMP2	5'- GCGACTGGGCCATGATTAGC -3’	5’- CCAGGGAGCAGTTGGCAAAG -3’
RAMP3	5’- CGCAACTTCTCCCGTTGCTG -3’	5’- GCGAAAGCCTTCCCACACAG -3’
CAIX	5’-AGTGCCTATGAGCAGTTGCT -3’	5’-TGCTTAGCACTCAGCATCAC -3’
RCP	5’-TGAGGTTCTTTGTCTGCTGGC -3’	5’- CGCAGAATTGGCATCCTTCAC-3’
Intermedin	5’-CGGCTTTGCACACGTAAAC-3’	5’- CCAGGGGGTCACAATTTCAG-3’

Primers used to screen for mutated clones obtained with CRISPR Cas9 procedure.

Genomic DNA amplification

5’- CGGAGGGAGATAAGCGTCT-3’	5’-AGCCCCCCCGAGTGGAAGTGC -3’

cDNA Amplification

5’- CCAGGGTCTGCGCTTCGCA -3’	5’- AGCCCCCCCGAGTGGAAGTGC-3’

References for the antibodies used in this study

ANTIBODY	MANUFACTURER	CATALOG NUMBER	DILUTION
ANTI-UCP1	Millipore	#662045	1000 (WB)
ANTI-UCP1	Abcam	AB10983	500 (ICC)
ANTI-Tubulin β1	SIGMA ALDRICH	#T7816	5000
ANTI-PLIN1	ACRIS Gmbh	#BP5015	1000
ANTI-p38 MAPK	Cell Signaling Technology	# 9212	1000
ANTI-phospho p38 MAPK	Cell Signaling Technology	#9211	1000
Anti E-Cadherin (24E10)	Cell Signaling Technology	#3195	500
Anti-human ADM	Phoenix Pharmaceuticals Inc	#H-010-28	500
Anti-human ADM	Santa Cruz Biotech	#SC 80462	500
Anti-phospho-HSL	Cell Signaling Technology	#4139	500
Anti-HSL	Cell Signaling Technology	#18381	500

Supplementary data: Table 1

References for the antibodies used in this study.

Supplementary Material and Methods:

hMADS cells differentiation.

Adipocyte differentiation of hMADS cells was carried out on confluent cultures. Cells were maintained in DMEM/Ham's F12 media (v/v) supplemented with 10 µg/ml transferrin, 0.85 µM insulin, 0.2 nM triiodothyronine, 1 µM dexamethasone (DEX), 100 µM isobutyl-methylxanthine and 1 µM rosiglitazone. 4 days later, the medium was changed (DEX and isobutyl-methylxanthine were omitted). The culture medium was then changed twice weekly. Neutral lipid accumulation was assessed by Oil red O staining.

ADM gene invalidation

Cells were transfected with plasmid expressing Cas9 nuclease and two guide RNAs targeting the *ADM* gene (supplementary Fig.7). The CRISPR kit used for constructing multiplex CRISPR/Cas9 vectors was a gift from Takashi Yamamoto (Addgene kit # 1000000055). Lipofectamine 2000 Reagent (Thermofisher) was used according to the manufacturer's protocol. Briefly, MCF-7 cells were seeded at 70% confluence in 12 well plates and cultured in growth medium without antibiotics for 24 h. The next day, cells were washed and transfected in serum-free OPTIMEM medium without antibiotics. Transfected cells were left one week for recovery and then seeded at one cell/well in 96 well plates. Genomic DNA was extracted from the growing clones using the Nucleospin DNA RapidLyse kit (Macherey Nagel). The CRISPR/Cas 9 procedure induced a 584 base pairs deletion in the genomic DNA accompanied by a shift in the open reading frame. PCR amplification of the genomic region targeted by the CRISPR/Cas 9 procedure gave a 535 bp PCR product for targeted clones and a 1119 bp amplicon for WT genomic DNA. The crude PCR product was purified and sequenced for 10 clones. The deletion was further confirmed at the RNA level, by RT-PCR. In this case, 366 bp or 569 bp amplicons were observed in modified or WT clones respectively.

Immunocytochemistry

Cells were rinsed twice with PBS, fixed with Roti-Histofix® (Roth, Lauterbourg, France) for 20 min at room temperature and permeabilized with triton X-100 (0.2% v/v) for 15 min. Blockade of non-specific sites was performed by incubation in PBS containing 4% of normal goat serum (NGS) for 30 min at room temperature. Cells were then incubated with the appropriate antibodies in the same buffer over night at 4°C. After 3 washes in PBS containing 0.4% of NGS, cover slips were incubated with the appropriate secondary antibody coupled to Alexa Fluor (1:1000) for 45 min at room temperature. Unspecific signal was evaluated for each antibody using a control condition without primary antibody and a non-specific antibody.

SUPPLEMENTARY FIGURES

Supplementary FIGURE 1

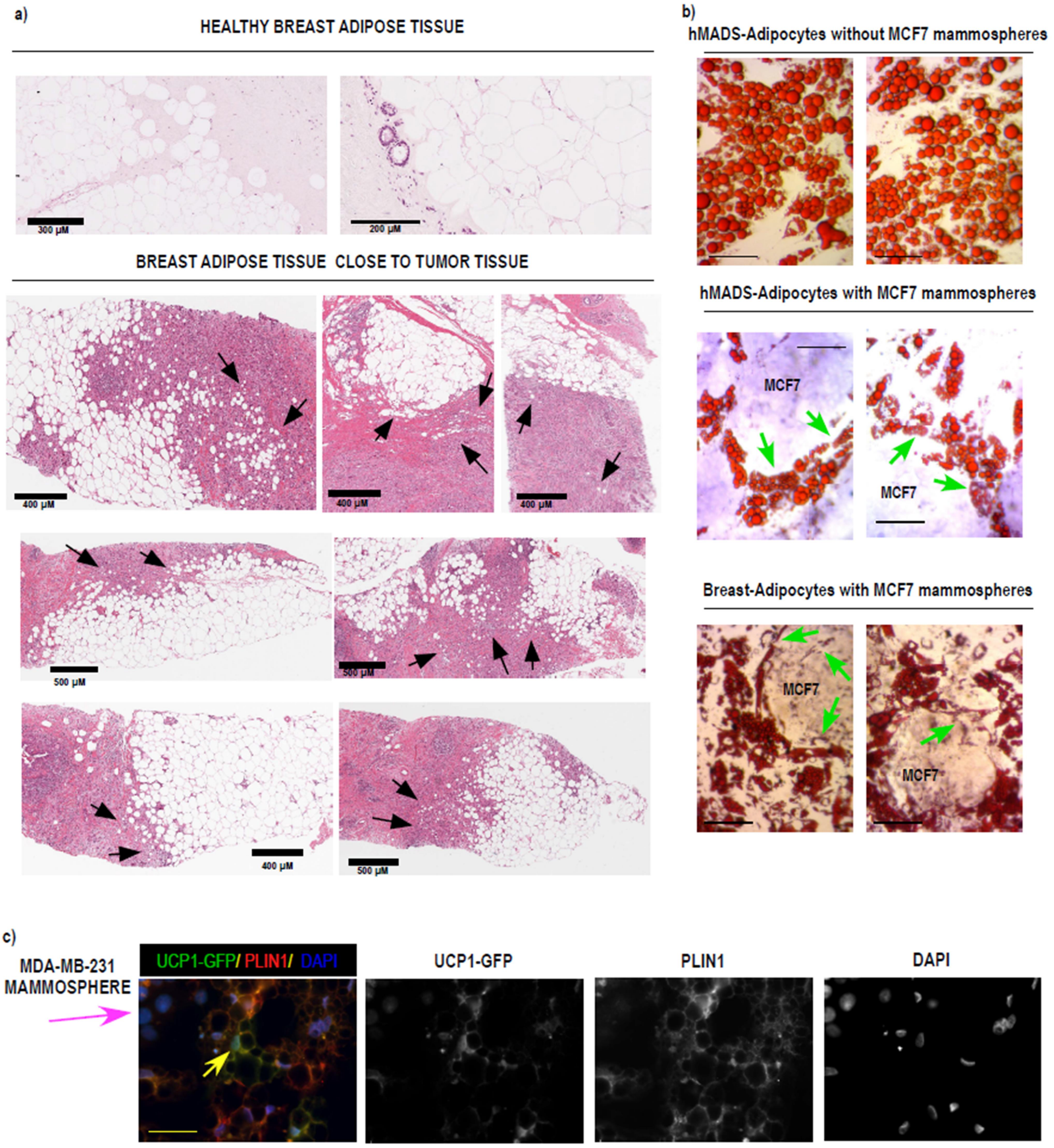

<u>Supplementary Figure 1:</u> <u>Morphology of adipocytes in contact with tumor cells or close to MCF7 mammospheres,</u> mammosphere-induced UCP1 expression in adipocytes.

a More histological sections of human healthy breast and breast tumor sections containing adipocytes are shown (revealed by hematoxylin and eosin staining). Adipocytes with small lipid droplets (black arrows) are preferentially observed close to the tumor cells, while those which are more distal display larger lipid droplets. (scale bar: as indicated in each panel).

b More pictures of the coculture of hMADS- and breast adipocytes with MCF7 mammospheres.

hMADS-adipocytes or breast-adipocytes were cultured in differentiation medium for 15 days. MCF7 cells were grown as mammospheres for 7 days. Co-culture of the 2 cell-types was performed for 2 days. Oil-Red O staining reveals that the size of the lipid droplets is heterogeneous, although smaller in adipocytes adjacent to the mammospheres (green arrows). Crystal violet was used to counterstain the MCF7 cells in presence of hMADS-adipocytes. Magnification X20, scale bar 100µM.

c MDA-MB-231 mammospheres induce GFP expression controlled by UCP1 promoter in hMADS-adipocytes.

MDA-MA-231 cells were grown as mammospheres for 7 days. They were co-cultured on a monolayer of hMADS cells expressing GFP under the control of UCP1 promoter that had been differentiated for 14 days. Co culture of the 2 cell types lasted for 4 days. PLIN1 (red, labelling the lipid droplets of adipocytes) expression was visualized using a specific antibody. GFP expression was visualized in green. DAPI was used to label the nuclei (Blue). The adipocytes adjacent to the mammospheres express both GFP and PLIN1. Magnification 40X. Scale bar 50µM.

Supplementary Figure 2

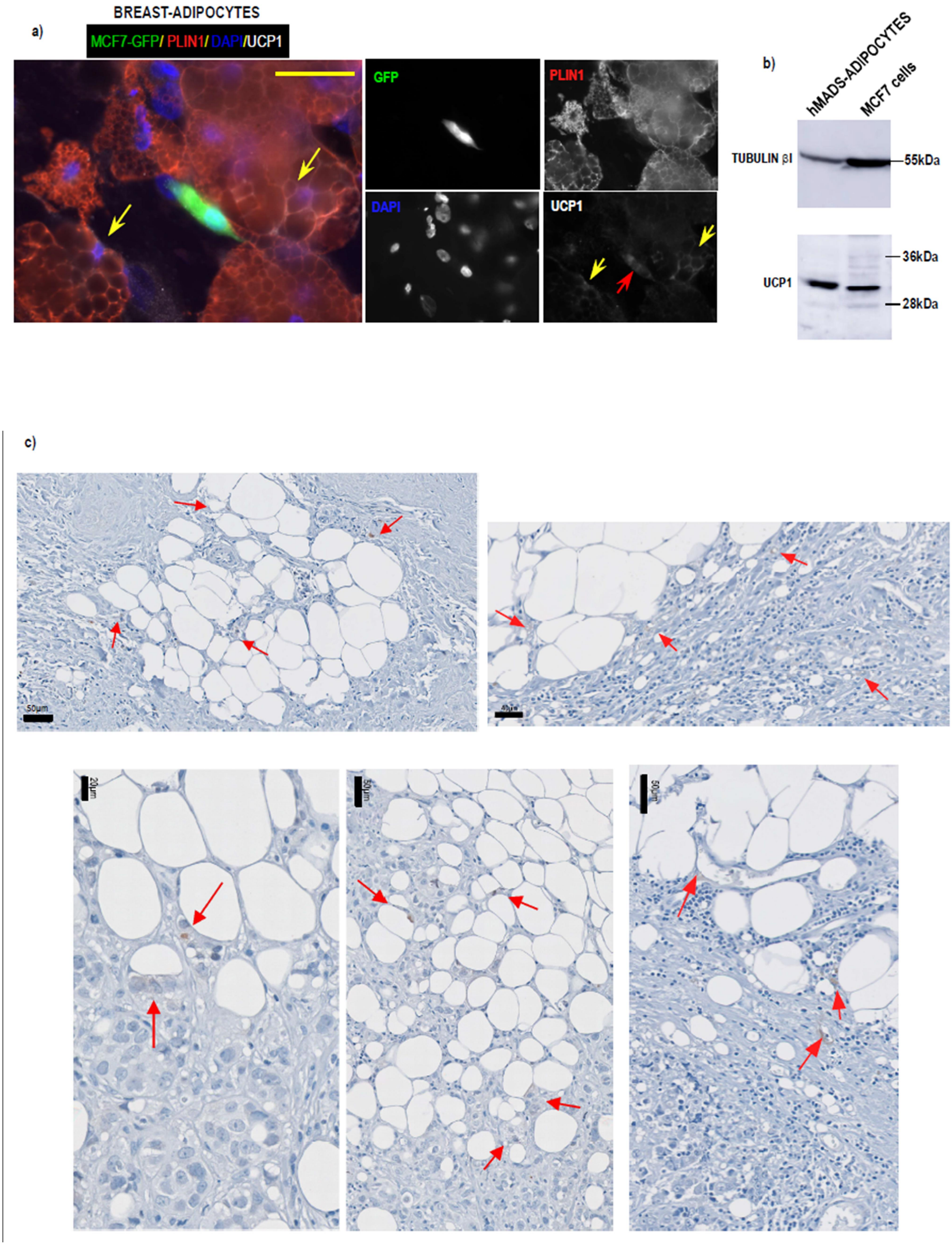

Supplementary Figure 2: MCF7 mammospheres induce UCP1 expression in breast-adipocytes

a) **MCF7 cells expressing GFP were grown as mammospheres for 7 days and co-cultured on a monolayer breast-adipocytes**. Co culture of the 2 cell types lasted for 4 days.

UCP1 (white) and PLIN1 (red) expressions were visualized using specific antibodies. DAPI was used to label the nuclei (Blue). The yellow arrows indicate UCP1 expression in adipocytes. Magnification 40X, scale bar 50µM. The recordings of fluorescence for GFP, PLIN1, UCP1and DAPI are shown in grey.

b). **UCP1 is detected in MCF7**

Expressions of Tubulin-βI used as a loading control (upper panel) and UCP1 (lower panel) were analyzed by Western blot using specific antibodies. Representative Western blots are shown. Full-length blots are presented in Supplementary Figure 9.

c) **UCP1 expression in patient breast tumor sections** was detected using immunohistochemical labeling with anti-UCP1 antibody (red arrows). The immunostains are shown in brown (scale bar: as indicated in each panel).

Supplementary Figure 3

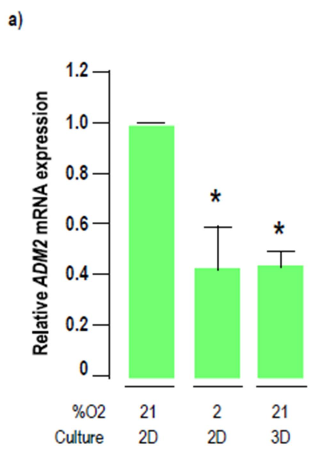

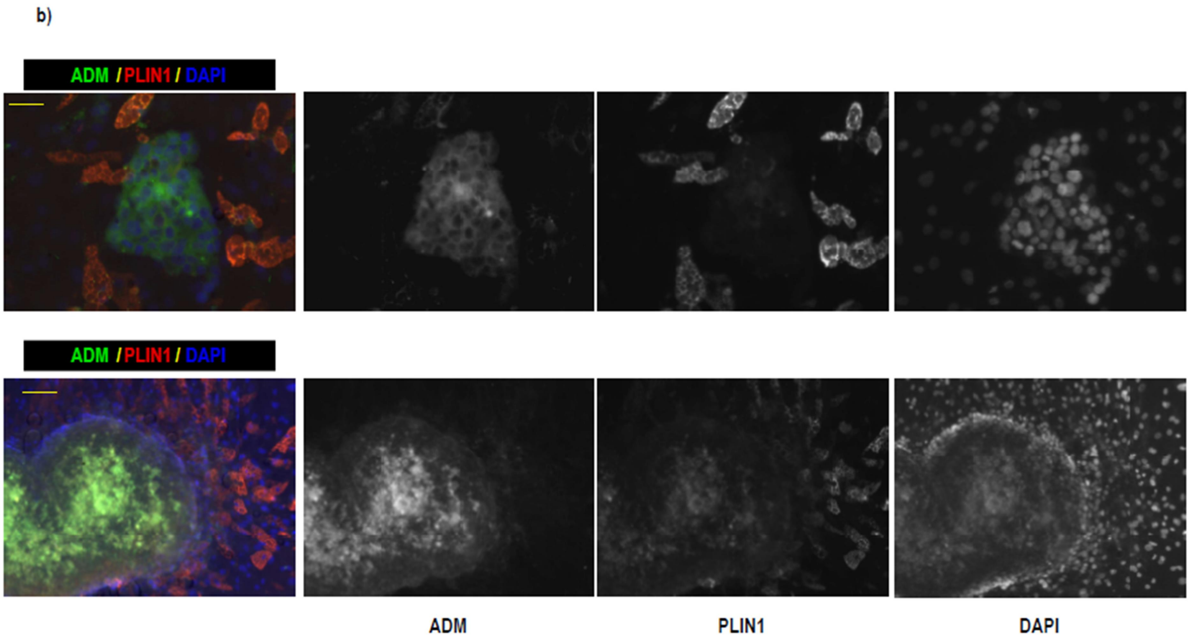

Supplementary Figure 3: ADM and intermedin expressions in MCF7 cells

a) MCF7 cells were grown as a monolayer under normoxia or hypoxia (2%O2) or mammospheres. Expression of intermedin was assessed by real-time RT-PCR and normalized for the expression of *36B4* mRNA. Histograms represent the mean of three independent experiments performed in duplicate. (*p<0.05).

b) MCF7 cells were grown as mammospheres for 7 days. Expressions of ADM (green) and PLIN1 (red) are shown. DAPI was used to label the nuclei (Blue). The fluorescence recorded for each channel is shown in separated pictures (see fig.3) (scale bar 50µm upper serie, 100 µM lower serie)

Supplementary Figure 4

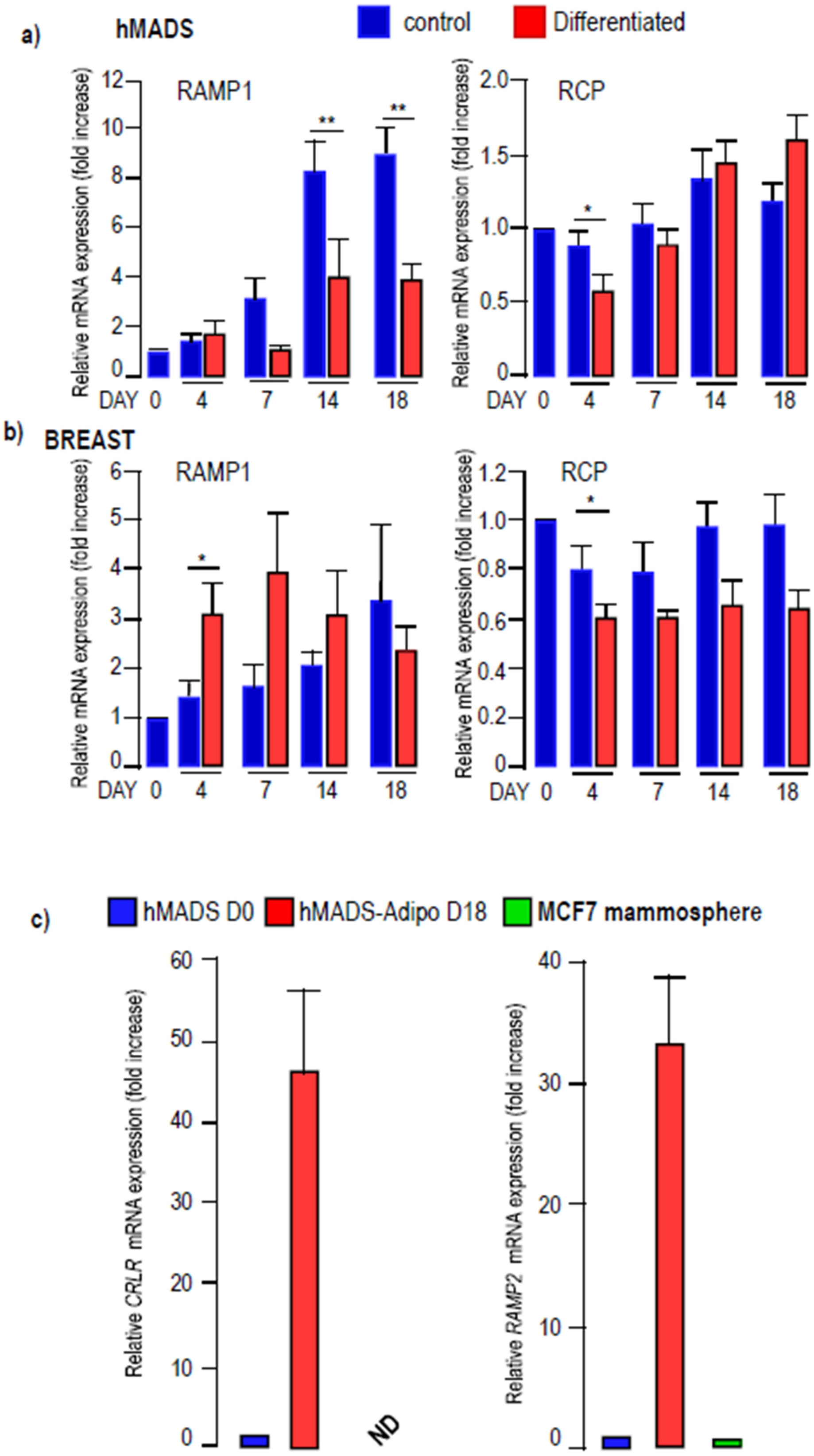

<u>Supplementary Figure 4:</u> **Expression of RAMP1 and RCP in hMADS and Breast-adipocytes and comparison of ADM receptors expression between hMADS-adipocytes and MCF7 mammospheres.**

a-b) **Time course for the expressions of *RAMP1 and RCP*** was assessed by real-time RT-PCR and normalized for the expression of *36B4* mRNA. Expressions were measured in cells that received (red bars) or did not receive (blue bars) the differentiation cocktail for the indicated number of days. The means ± SEM were calculated from five 5 independent experiments, with determinations performed in duplicate (*$p<0.05$, ** $p<0.01$).

c) **Expressions of *CRLR* and *RAMP2*** were assessed by real-time RT-PCR and normalized for the expression of *36B4* mRNA. They were measured in cells that received (red bars) or not (blue bars) the differentiation cocktail for 18 days or in MCF7 mammospheres (green bars). ND means not detected.

Supplementary Figure 5

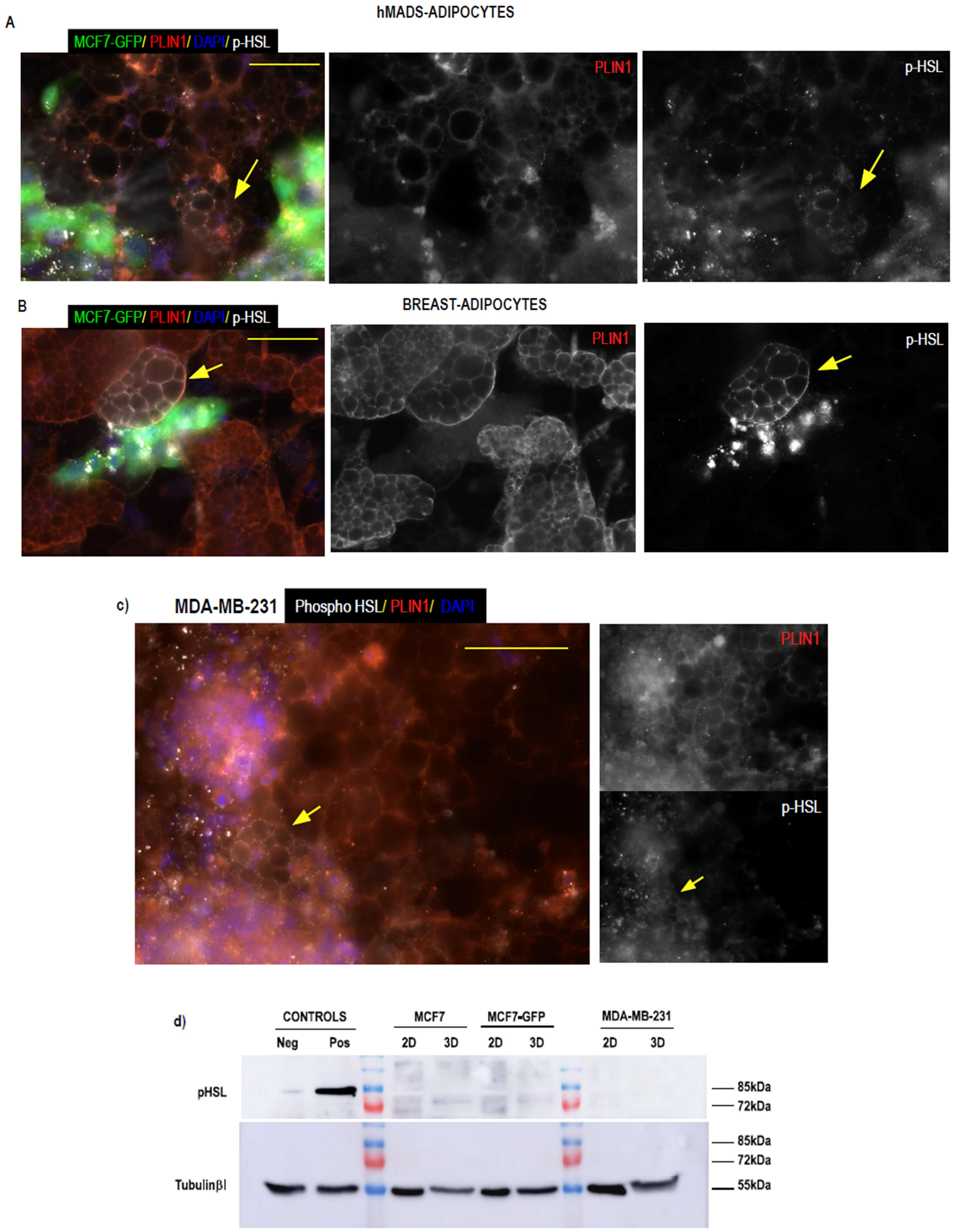

<u>Supplementary Figure 5:</u> MCF7 and MDA-MB-231 mammospheres induced phosphorylation of HSL in hMADS- adipocytes and breast-adipocytes close to the mammospheres.

a-b-c) MCF7-GFP or MDA-MB-231 cells were grown as mammospheres for 7 days and co-cultured on a monolayer of hMADS cells differentiated for 14 days or breast-adipocytes differentiated for 21 days. Co culture of the 2 cell types lasted for 4 days.

Phosphorylation of HSL (white) and PLIN1 (red) expressions were visualized using specific antibodies. DAPI was used to label the nuclei (Blue). The yellow arrows indicate phospho- HSL expression. Magnification 40X, scale bar 50µM. The recording of the channels for PLIN1 and phospho-HSL are shown in grey.

d) Absence of pHSL in mammospheres,

Protein expression was measured in MCF7, MCF7_GFP or MDA-MB-231 cells grown in 2 dimension (2D) or as mammospheres (3D). 30 µg of proteins were loaded in each lane. Controls to monitor pHSL expression were loaded on the same gel (Negative control: hMADS-adipocytes, positive control: hMADS- adipocytes stimulated with 10µM forskolin for 4 hours (20 µg of proteins were loaded). Expressions of pHSL (upper panel) and Tubulin-βI (lower panel) used as a loading control were analyzed by Western blot using specific antibodies. Representative Western blots are shown. Full-length blots are presented in Supplementary Figure 9.

Supplementary Figure 6

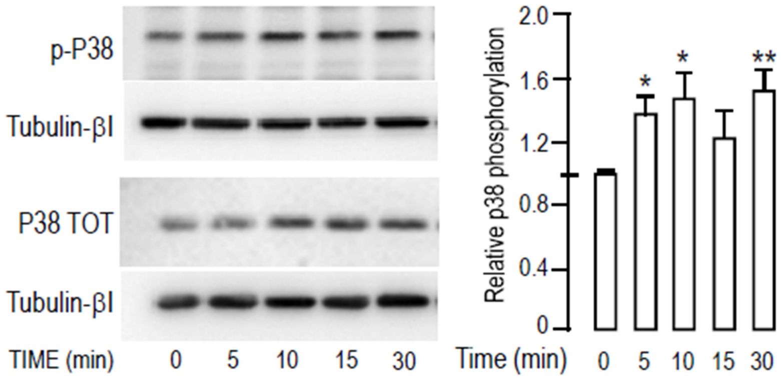

Supplementary Figure 6: ADM induces phosphorylation of p38 MAPK.

a, ADM increased phosphorylation of p38 MAPK.

Protein expression and phosphorylation were measured in hMADS-adipocytes grown in the differentiation medium. They were stimulated by ADM for the indicated time. Expressions of P38 MAPK (upper panel) and Tubulin-βI (lower panel) used as a loading control as well as total P38 MAPK were analyzed by Western blot using specific antibodies. Representative Western blots are shown. Full-length blots are presented in Supplementary Figure 9.

b, Quantification of the signals.

Expression of the proteins was quantified using the Quantity one Program and compared to the expression of Tubulin-βI. The means ± SEM were calculated from five independent experiments (*p<0.05, ** p<0.01).

SUPPLEMENTARY FIGURE 7

Analysis of the sequences of the MCF7 clones obtained after ADM invalidation.

a / Localization of the guide RNAs to target the *ADM* gene (genomic sequence). The introns are in black and the exons within the translated part of the sequence are in blue capital letters.

The localizations of the guide RNAs are underlined in yellow and in pink.

GGCCGGCGCGTGCAAACGCCTCTGGCGCCTCTCTGCGCGGAGGGAGATAAGCGTCTGAGCCAGGGAAAGCGCGG
GCTAAACCCGCCTCGCCGGGGCCCCTGCCCGCCCTCCGTGCCCCGCCCGGGCGGTGCAGCTGGCCCGGGTGCTC
ACGCTCGACTCTCTTTCTTCTTTTCCAGGGTCTGCGCTTCGCAGCCGGG**ATG**AAGCTGGTTTCCGTCGCCCTGATGT
ACCTGGGTTCGCTCGCCTTCCTAGGCGCTGACACCGCTC/GGTTGGATGTCGCGTCGGAGTTTCGAAAGAAgtgagtccg
ggcagcgccttccccttgctggtacctggcaggcaaggggaactgaccgttggtcccgaaggtctagaagtgaatgggagcagggacaggcctgggcgtcacctgaac
gcacgcgaatcgggtctgcttgtgttttccagGTGGAATAAGTGGGCTCTGAGTCGTGGGAAGAGGGAACTGCGGATGTCCAGCAGC
TACCCCACCGGGCTCGCTGACGTGAAGGCCGGGCCTGCCCAGACCCTTATTCGGCCCCAGGACATGAAGGGTGCC
TCTCGAAGCCCCGAAGACAGgtaactacgccctgtgctgtccagggacgggagggaaggaaggtgtgcgggaggagttctctgtctccactcccctggccc
gggggatcgtcggggctggaccgcagctcagatggcgcgagcagtttccagctccctctggctctagaatggctcccgttcccggtgttggggccaaagctctgcttgatgg
ggtctcaagttgcctttcttcccctccccccgcccgcagCAGTCCGGATGCCGCCCGCA/TCCGAGTCAAGCGCTACCGCCAGAGCATG
AACAACTTCCAGGGCCTCCGGAGCTTTGGCTGCCGCTTCGGGACGTGCACGGTGCAGAAGCTGGCACACCAGATCT
ACCAGTTCACAGATAAGGACAAGGACAACGTCGCCCCCAGGAGCAAGATCAGCCCCCAGGGCTACGGCCGCCGGC
GCCGGCGCTCCCTGCCCGAGGCCGGCCCGGGTCGGACTCTGGTGTCTTCTAAGCCACAAGCACACGGGGCTCCAG
CCCCCCCGAGTGGAAGTGCTCCCCACTTTCTT**TAG**GATTTAGGCGCCCATGGTACAAGGAATAGTCGCGCAAGCAT

b/ PCR identification of the clones obtained.

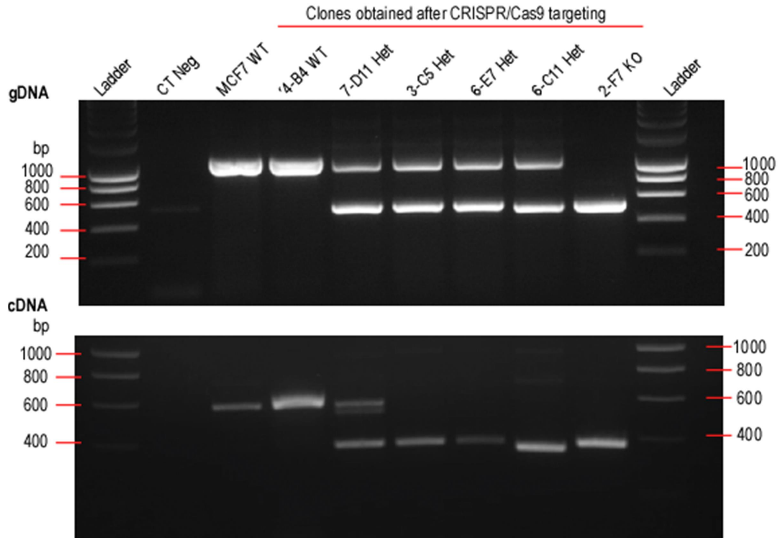

c/ Sequences of the sequenced PCR products covering the ADM genomic region targeted by the CRISPR gRNA in clone 2F7 showing the 2 mutated alleles.

Nucleotide alignment compared to WT sequence

```
ADM-Wt          ATGAAGCTGGTTTCCGTCGCCCTGATGTACCTGGGTTCGCTCGCCTTCCTAGGCGCTGAC
Clone 2F7       ATGAAGCTGGTTTCCGTCGCCCTGATGTACCTGGGTTCGCTCGCCTTCCTAGGCGCTGAC
Clone 6C11      ATGAAGCTGGTTTCCGTCGCCCTGATGTACCTGGGTTCGCTCGCCTT
                ***********************************************

ADM-Wt          ACCGCTCGGTTGGATGTCGCGTCGGAGTTTCGAAAGAAGTGAGTCCGGGCAGCGCCTTCC
Clone 2F7       ACCGCTCG----------------------------------------------------
Clone 6C11      ------------------------------------------------------------

ADM-Wt          CCCTTGCTGGTACCTGGCAGGCAAGGGGAACTGACCGTTGGTCCCGAAGGTCTAGAAGTG
Clone 2F7       ------------------------------------------------------------
Clone 6C11      ------------------------------------------------------------

ADM-Wt          AATGGGAGCAGGGACAGGCCTGGGCGTCACCTGAACGCACGCGAATCGGGTCTGCTTGTG
Clone 2F7       ------------------------------------------------------------
Clone 6C11      ------------------------------------------------------------

ADM-Wt          TTTTCCAGGTGGAATAAGTGGGCTCTGAGTCGTGGGAAGAGGGAACTGCGGATGTCCAGC
Clone 2F7       ------------------------------------------------------------
Clone 6C11      ------------------------------------------------------------

ADM-Wt          AGCTACCCCACCGGGCTCGCTGACGTGAAGGCCGGGCCTGCCCAGACCCTTATTCGGCCC
Clone 2F7       ------------------------------------------------------------
Clone 6C11      ------------------------------------------------------------

ADM-Wt          CAGGACATGAAGGGTGCCTCTCGAAGCCCCGAAGACAGGTAACTACGCCCTGTGCTGTCC
Clone 2F7       ------------------------------------------------------------
Clone 6C11      ------------------------------------------------------------

ADM-Wt          AGGGACGGGAGGGAAGGAAGGTGTGCGGGAGGAGTTCTCTGTCTCCACTCCCCTGGCCCG
Clone 2F7       ------------------------------------------------------------
Clone 6C11      ------------------------------------------------------------

ADM-Wt          GGGGATCGTCGGGGCTGGACCGCAGCTCAGATGGCGCGAGCAGTTTCCAGCTCCCTCTGG
Clone 2F7       ------------------------------------------------------------
Clone 6C11      ------------------------------------------------------------

ADM-Wt          CTCTAGAATGGCTCCCGTTCCCGGTGTTGGGGCCAAAGCTCTGCTTGATGGGGTCTCAAG
Clone 2F7       ------------------------------------------------------------
Clone 6C11      ------------------------------------------------------------

ADM-Wt          TTGCCTTTCTTCCCCCTCCCCCCGCCCGCAGCAGTCCGGATGCCGCCCGCATCCGAGTCA
Clone 2F7       ---------------------------------------------------TCCGAGTCA
Clone 6C11      ---------------------------------------------------TCCGAGTCA
                                                                   *********

ADM-Wt          AGCGCTACCGCCAGAGCATGAACAACTTCCAGGGCCTCCGGAGCTTTGGCTGCCGCTTCG
Clone 2F7       AGCGCTACCGCCAGAGCATGAACAACTTCCAGGGCCTCCGGAGCTTTGGCTGCCGCTTCG
Clone 6C11      AGCGCTACCGCCAGAGCATGAACAACTTCCAGGGCCTCCGGAGCTTTGGCTGCCGCTTCG
                ************************************************************

ADM-Wt          GGACGTGCACGGTGCAGAAGCTGGCACACCAGATCTACCAGTTCACAGATAAGGACAAGG
Clone 2F7       GGACGTGCACGGTGCAGAAGC---------------------------------------
Clone 6C11      GGACGTGCACGGTGCAGAAGCTGGCACACCAGATCTACCAGTTCACAGATAAGGACAAGG
                *********************
```

d/ Pre-pro adrenomedullin protein sequence alignment of WT and mutated clones of ADM.

```
WT              MKLVSVALMYLGSLAFLGADTARLDVASEFRKKWNKWALSRGKRELRMSSSYPTGLADVK 60
2F7             MKLVSVALMYLGSLAFLGADTAR------------------------PSQALPP-EHEQL 35
6C11            MKLVSVALMYLGSLAF-------------------------------PSQALPP-EHEQL 28
                ****************                                *.: *.   :

WT              AGPAQTLIRPQDMKGASRSPEDSSPDAARIRVKRYRQSMNNFQGLRSFGCRFGTCTVQKL 120
2F7             PGPPELWLPLRDVHGAEAGTPDLP--VHR*------------------------------ 62
6C11            PGPPELWLPLRDVHGAEAGTPDLP--VHR*----------------------------- 55
                .**.:  :  :*::**. .. * .  . *

WT              AHQIYQFTDKDKDNVAPRSKISPQGYGRRRRRSLPEAGPGRTLVSSKPQAHGAPAPPSGS 180
2F7             ------------------------------------------------------------
6C11            ------------------------------------------------------------

WT              APHFL 185
2F7             -----
6C11            -----
```

LEGEND

Signal peptide

PAMP peptide

ADM peptide

Clivage sites **KR** & **RR**

MR-Pro ADM: inactive peptide

Supplementary Figure 7: Analysis of the sequences of the MCF7 clones obtained after ADM invalidation.

a/ Localization of the guide RNAs to target the ADM gene. Sequence of the ADM gene: The introns are in black and the exons in the translated part of the gene are in blue capitals. Localizations of the two guide RNAs are highlighted in yellow and pink.

b/ PCR identification of the clones obtained.

a-b) Clones were analysed as described in material and methods. The presence of WT or mutated alleles was measured by PCR from genomic DNA or reverse transcriptase polymerase chain reaction (RT-PCR) when analysed from RNA. For genomic DNA analysis: the 1119 base pairs band corresponds to the WT allele and the 535 bp band corresponds the mutated allele. For RNA analysis: a 569 base pairs band corresponds to the WT allele and a 366 bp band corresponds the mutated allele. Note that most of the heterozygous clones display a monoallelic expression of the mutated allele.

c/ Alignment of the wild type and mutated clones.

Sequence is shown for clone 2F7 which corresponds to the ADM-KO clone. All mutated clones displayed the same sequence except the 6-C-11 clone.

d/ Alignment of the proteins produced after translation.

The sequence of pre-pro ADM is shown and the highlighted zones indicate the position of the signal peptide, PAMP peptide and ADM peptide. The cleavage sites are indicated in red. None of the clones was able to produce functional full length pre-pro-ADM as the shift in the open reading frame modified the ADM sequence. In addition, all cleavage sites to produce a functional ADM peptide were lost.

Supplementary Figure 8

(PART1)

Single channel images for Figure 6a

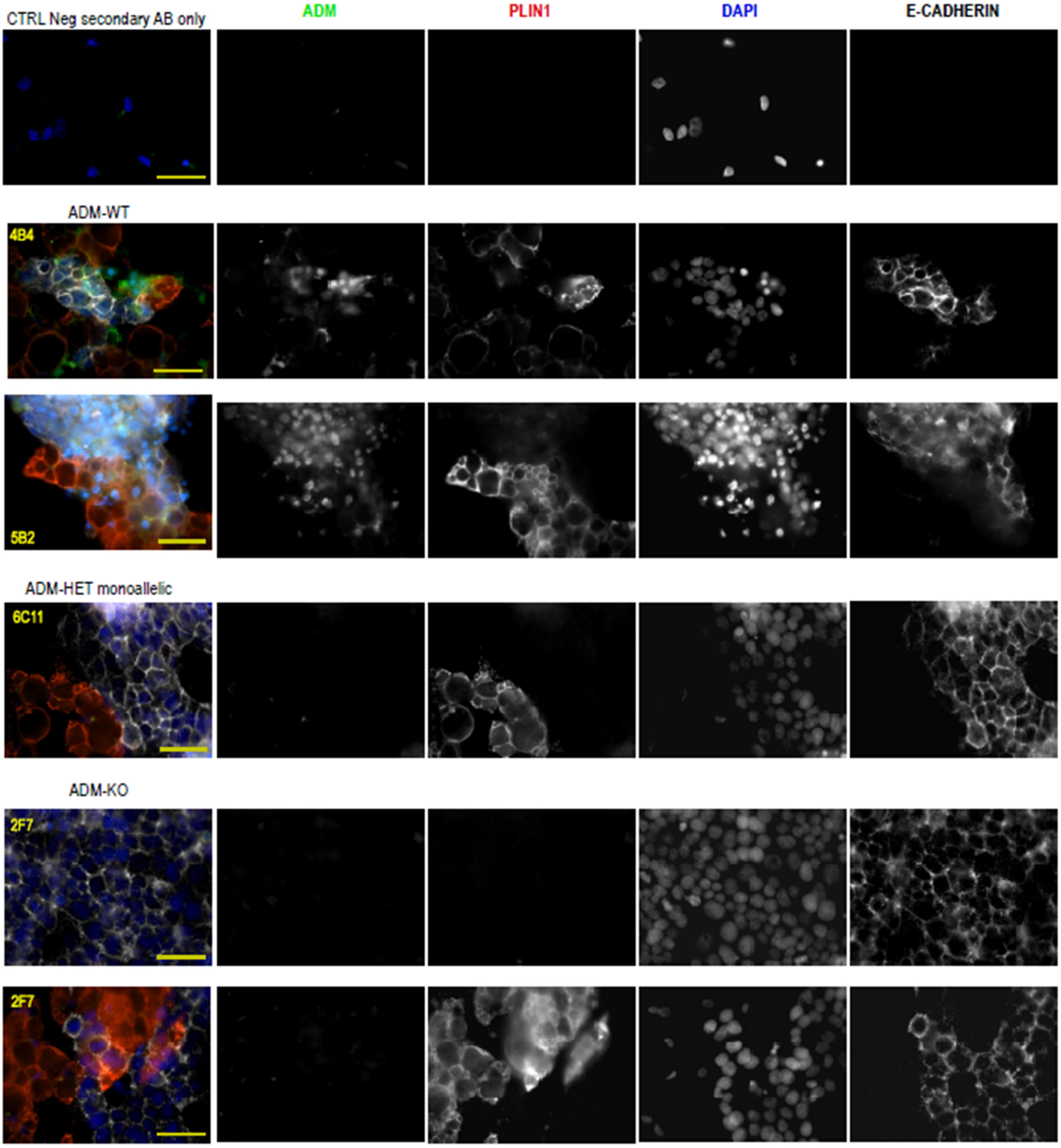

Supplementary Figure 8

(PART2)

Single channel images for Figure 6b

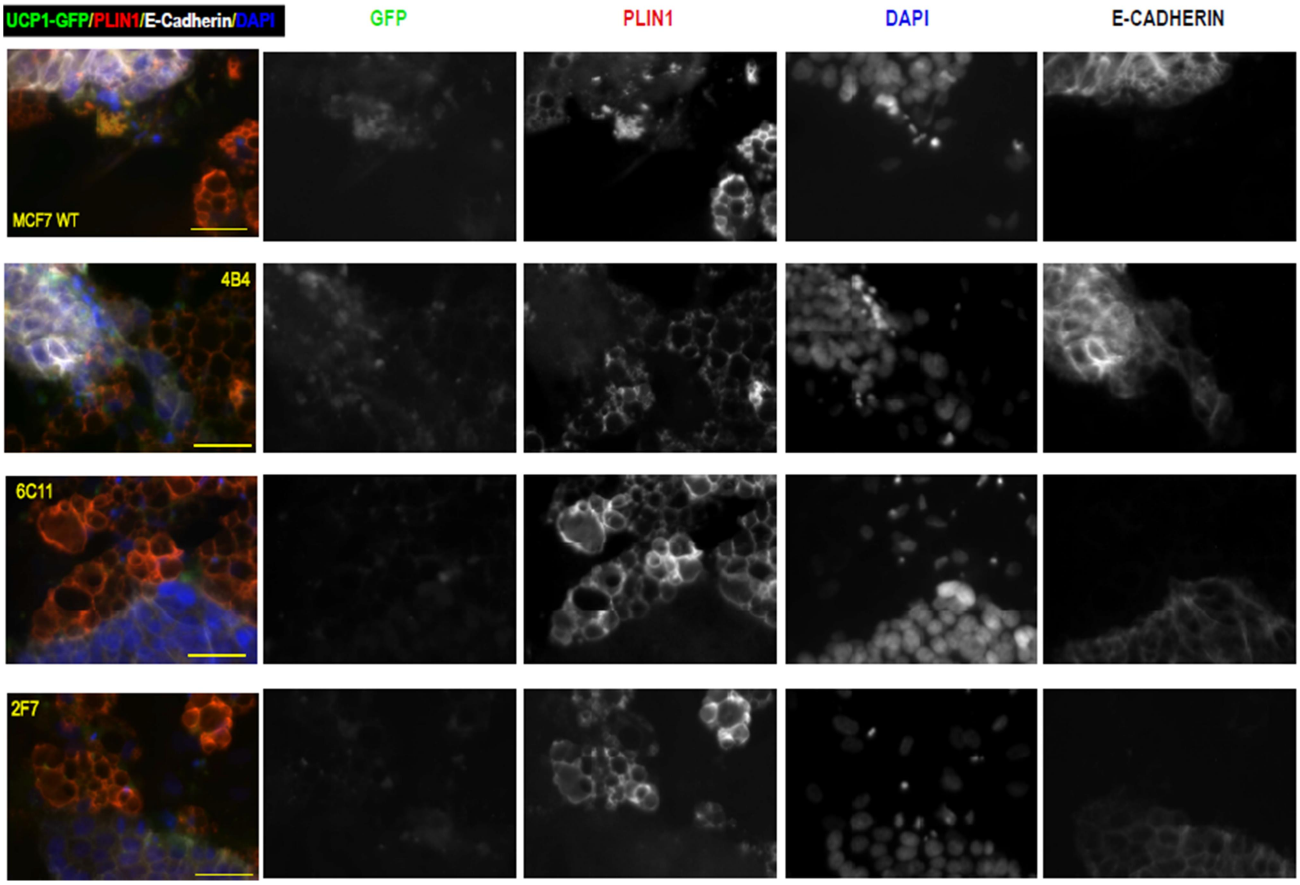

Supplementary Figure 8

(PART3)

Single channel images for Figure 6d

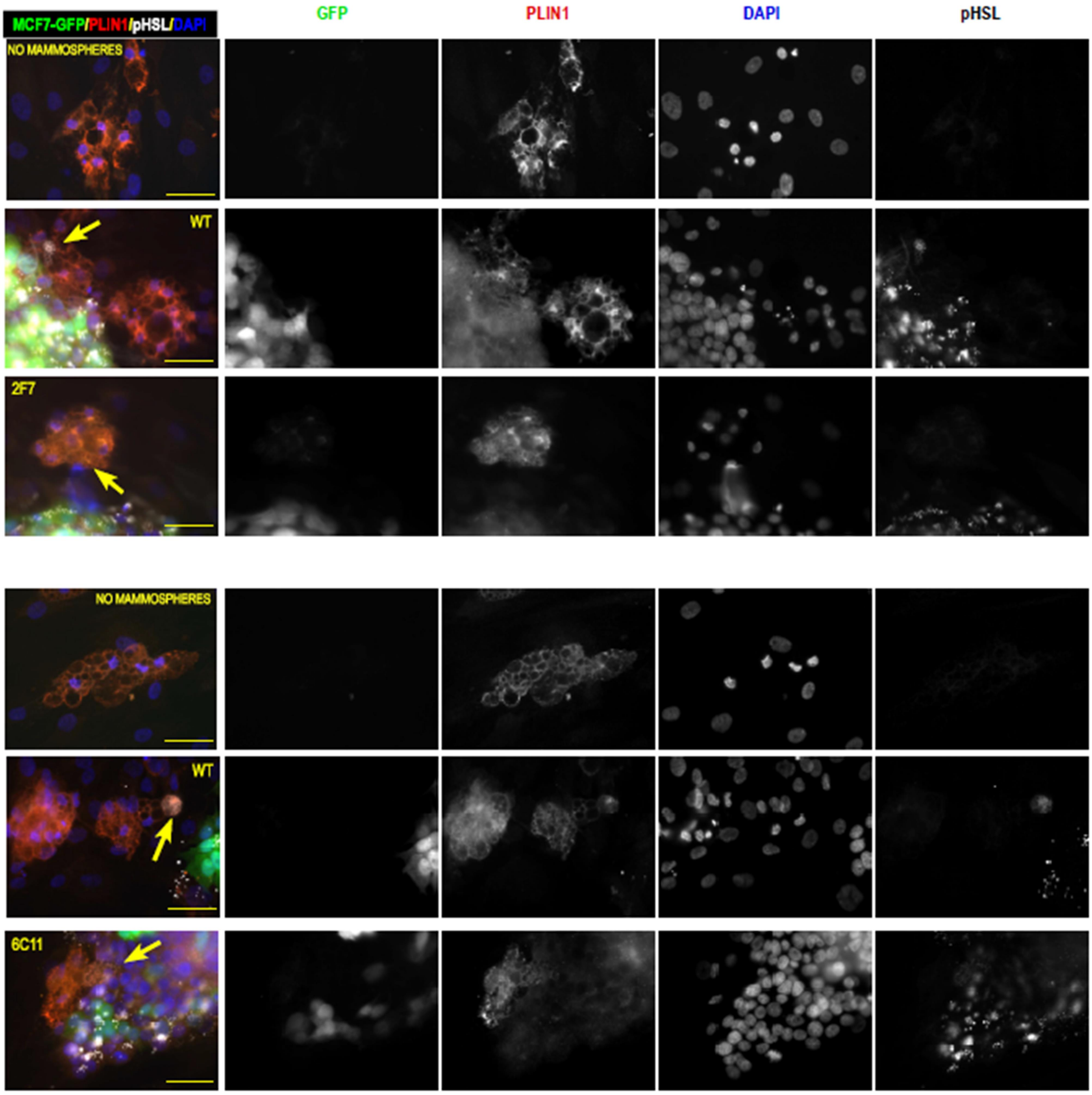

Supplementary Figure 9

This figure shows the full-length blots.

SUPPLEMENTARY FIGURE 9 (PART 1)

Original images for figure 4

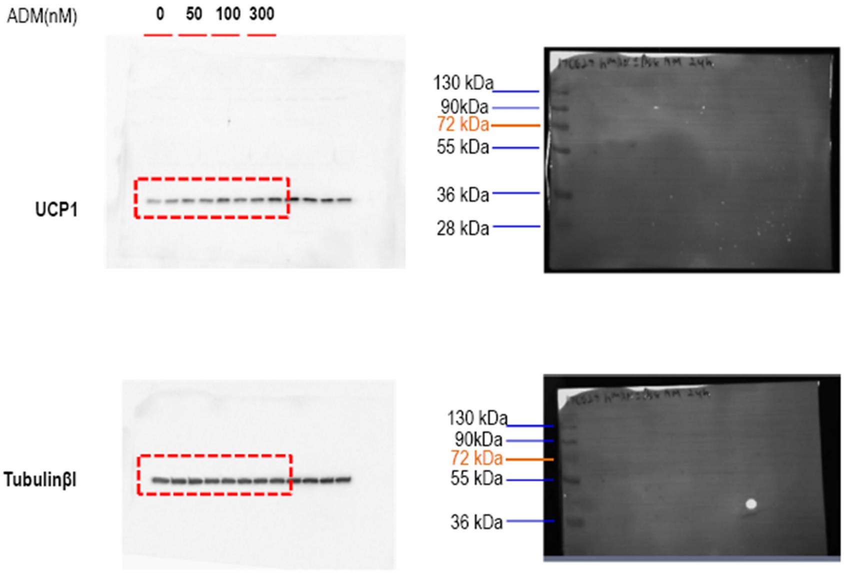

SUPPLEMENTARY FIGURE 9 (PART 2)

Original images for figure 5

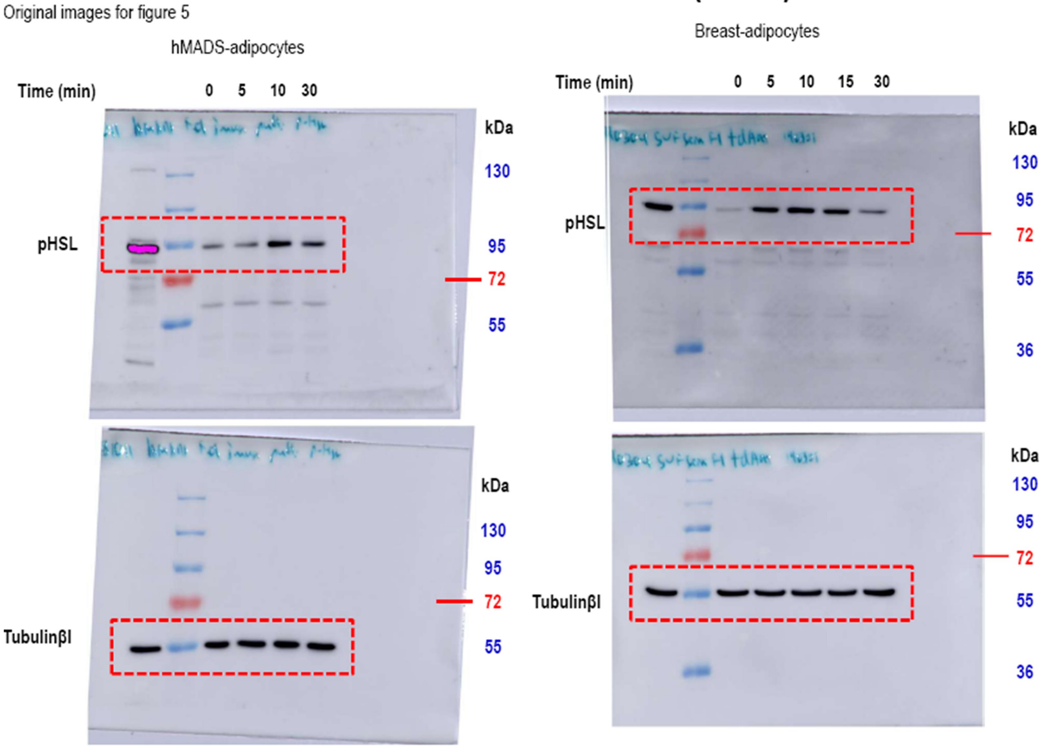

SUPPLEMENTARY FIGURE 9 (PART 3)

Original images for Supplementary figure 2

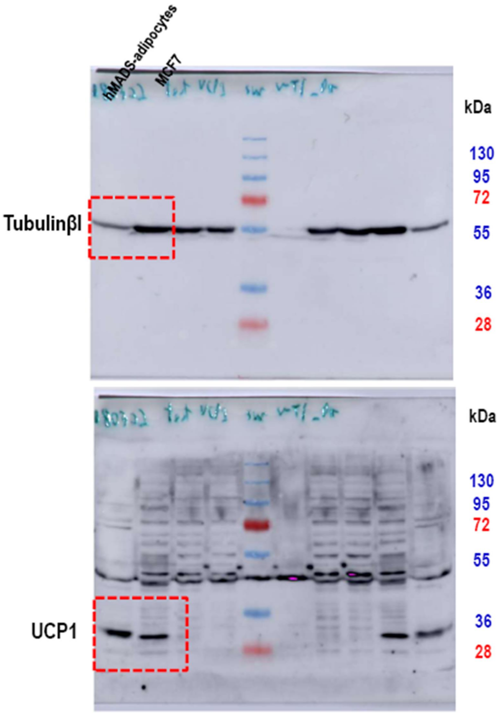

Original images for Supplementary figure 5

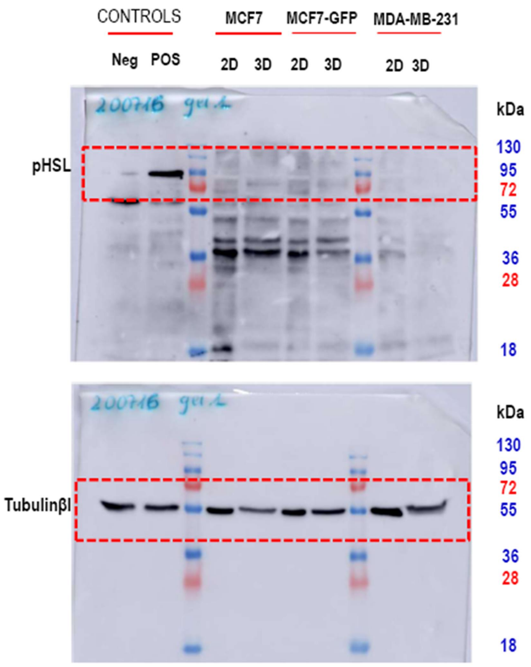

SUPPLEMENTARY FIGURE 9 (PART4)

Original images for Supplementary figure 6

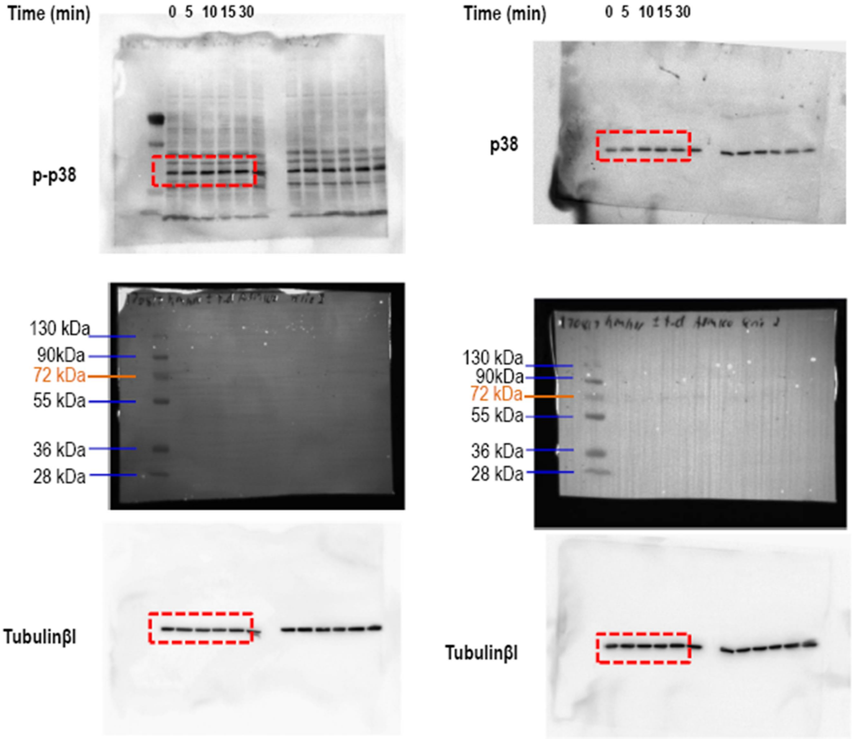

Supplement: Supplementary file 1 — Additional file 1. [file 12885_2020_7273_MOESM1_ESM.pdf]
